# Co-occurrence of ecologically similar species of Hawaiian spiders reveals critical early phase of adaptive radiation

**DOI:** 10.1186/s12862-018-1209-y

**Published:** 2018-06-19

**Authors:** Darko D. Cotoras, Ke Bi, Michael S. Brewer, David R. Lindberg, Stefan Prost, Rosemary G. Gillespie

**Affiliations:** 1 Department of Integrative Biology, University of California, 3060 Valley Life Sciences Building, Berkeley, CA 94720-3140 USA; 2 Museum of Vertebrate Zoology, University of California, 3101 Valley Life Sciences Building, Berkeley, CA 94720-3160 USA; 3 Computational Genomics Resource Laboratory (CGRL), California Institute for Quantitative Biosciences (QB3), University of California, Berkeley, CA 94720-3102 USA; 4 Department of Biology, East Carolina University, 1000 E 5th St, Greenville, NC 27858-4353 USA; 5 Museum of Paleontology, University of California, 1101 Valley Life Sciences Building, Berkeley, CA 94720 USA; 6 Department of Biology, Stanford University, Stanford, CA 94305-5020 USA; 7 Department of Environmental Science, University of California, 130 Mulford Hall, Berkeley, CA 94720-3114 USA; 8 Department of Ecology & Evolutionary Biology, University of California Santa Cruz, Santa Cruz, CA 95064 USA; 9 Department of Entomology / Center for Comparative Genomics, California Academy of Sciences, San Francisco, CA 94118 USA

**Keywords:** *Tetragnatha*, Ecomorph, Exon capture, Phylogeography

## Abstract

**Background:**

The processes through which populations originate and diversify ecologically in the initial stages of adaptive radiation are little understood because we lack information on critical steps of early divergence. A key question is, at what point do closely related species interact, setting the stage for competition and ecological specialization? The Hawaiian Islands provide an ideal system to explore the early stages of adaptive radiation because the islands span ages from 0.5–5 Mya. Hawaiian spiders in the genus *Tetragnatha* have undergone adaptive radiation, with one lineage (“spiny legs”) showing four different ecomorphs (*green, maroon, large brown, small brown*); one representative of each ecomorph is generally found at any site on the older islands. Given that the early stages of adaptive radiation are characterized by allopatric divergence between populations of the same ecomorph, the question is, what are the steps towards subsequent co-occurrence of different ecomorphs? Using a transcriptome-based exon capture approach, we focus on early divergence among close relatives of the *green* ecomorph to understand processes associated with co-occurrence within the same ecomorph at the early stages of adaptive radiation.

**Results:**

The major outcomes from the current study are first that closely related species within the same *green* ecomorph of spiny leg *Tetragnatha* co-occur on the same single volcano on East Maui, and second that there is no evidence of genetic admixture between these ecologically equivalent species. Further, that multiple genetic lineages exist on a single volcano on Maui suggests that there are no inherent dispersal barriers and that the observed limited distribution of taxa reflects competitive exclusion.

**Conclusions:**

The observation of co-occurrence of ecologically equivalent species on the young volcano of Maui provides a missing link in the process of adaptive radiation between the point when recently divergent species of the same ecomorph occur in allopatry, to the point where different ecomorphs co-occur at a site, as found throughout the older islands. More importantly, the ability of close relatives of the same ecomorph to interact, without admixture, may provide the conditions necessary for ecological divergence and independent evolution of ecomorphs associated with adaptive radiation.

**Electronic supplementary material:**

The online version of this article (10.1186/s12862-018-1209-y) contains supplementary material, which is available to authorized users.

## Background

**Fig. 1:**
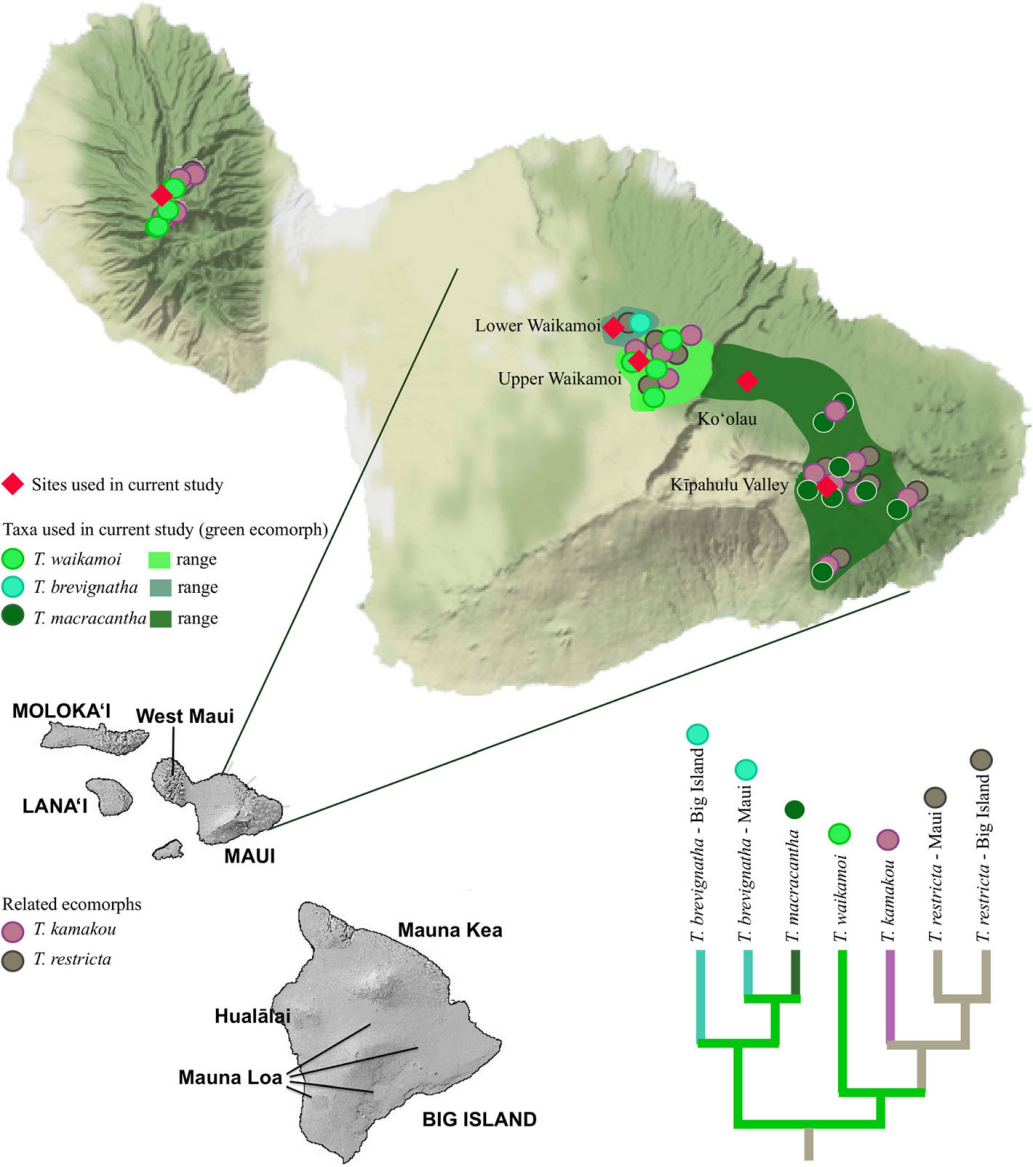
Distributions of the Maui representatives of the *green* ecomorph and other ecomorphs in the clade. The map shows the distributions of the three *green* ecomorph species based on previous literature and field observations. The three species have populations on different volcanoes as follows: *T, macracantha* (dark green), Kīpahulu Valley (East Maui) and Lanaʻi; *T. waikamoi* (light green), Upper Waikamoi (East Maui) and West Maui (Puʻu Kukui); *T. brevignatha* (capypso), Lower Waikamoi (East Maui) and the Big Island (Mauna Kea - Laupāhoehoe; Mauna Loa - Kīpuka, Kaʻu, Kona Hema, and Puʻu Makaʻala; and Hualālai – Honoaula; localities not shown on the figure). Circles show a reduced representation of previous collections of species within the *green* ecomorph, as well as those from the maroon (*T. kamakou*) and small brown (*T. restricta*) ecomorphs. Red diamonds correspond to the sites used on the current study; for more details see Additional file 1: Table S1. The cladogram indicates the phylogenetic position of species used in the current study relative to others within the spiny leg clade (based on [11, 22]), the colours again representing the ecomorph of each species (for details see [11]). Maps modified from http://www.hear.org/starr/maps/stock/ and Stamen terrain map

Adaptive radiation is the rapid diversification of an ancestral species into several ecologically different species, associated with adaptive morphological or physiological divergence [1]. The phenomenon involves the interplay of ecological and evolutionary processes, and as such is central to understanding mechanisms of evolution through natural selection. Because most cases of adaptive radiation in nature necessarily unfold over extended time periods, comparative inference is often the only way to infer evolutionary and ecological underpinnings. As a consequence, the mechanism through which diversity originates in the course of adaptive radiation is very poorly understood. In particular, where multiple close relatives co-occur within a rapidly multiplying lineage, the processes that characterize early differentiation are almost entirely unknown. In terms of species accumulation alone, this may occur through the joint processes of immigration and speciation [2], with successful establishment the result of differing mechanisms of competitive exclusion [3] on the one hand acting to limit access of similar species to a site, and ecological character displacement [4] on the other, in which traits evolve through selection acting to reduce resource competition between species. However, recent studies have highlighted the importance of “nonadaptive” [5] or minimally adaptive [6] divergence in the early stages of adaptive radiation. The current study sets out to elucidate the processes involved in the early stages of adaptive radiation in a lineage of long-jawed spiders (genus *Tetragnatha*) within the Hawaiian Islands in which diversification has led to the presence of non-random, parallel sets of ecological forms, or ecomorphs [7]. Specifically, taxa within the monophyletic “spiny leg” clade exhibit 4 discrete ecological forms that co-occur in almost every site in the Hawaiian Islands (Fig. 1), while species of the same ecomorph are largely allopatric [7]. The question then is, if early diverging taxa are inevitably the same ecomorph and allopatric, then what might be the selective pressure for divergence, and subsequent co-occurrence, of different ecomorphs? In particular, can, and if so when do, closely related species of the same ecomorph co-occur in the process, providing the opportunity for competition and ecological specialization? Here we focus explicitly on the early divergence within close relatives of the *green spiny* form to assess evidence for co-occurrence within the same ecomorph at the early stages of adaptive radiation, and the possible outcomes of co-occurrence events, whether competitive exclusion, hybridization and genetic admixture, or simply haphazard accumulation of genetic diversity within the ecomorph.

### The Hawaiian Islands

The Hawaiian archipelago is the product of a volcanic hotspot, well known for some of the most extraordinary examples of adaptive radiations [8]. The biogeographic pattern that predominates in most Hawaiian taxa, both at the species and population level, is a step-like progression down the island chain from the oldest to the youngest islands [8], often with repeated bouts of diversification within islands [9]. Accordingly, the islands are considered a “natural laboratory” as they allow study of patterns of species formation across snapshots of evolutionary time [10, 11]. The current study is limited to the youngest islands of the archipelago, Maui (< 2 Mya), Lanaʻi (< 1.5 Mya) and Big Island (< 0.5 Mya), where genetic entities appear to accumulate most rapidly in most lineages [10, 12, 13].

### Tetragnatha ecomorphs

Long-jawed orb-weaving spider genus *Tetragnatha* (Tetragnathidae) have undergone adaptive radiation in the Hawaiian Islands, with the early stages of the process played out on the youngest islands [11]. As a whole, the genus *Tetragnatha* has approximately 360 described species with worldwide distribution, generally characterized by a light and fragile orb web built over water or in other wet places [14]. The striking diversity in the Hawaiian Islands, with multiple co-occurring species throughout the islands, stands in contrast to its global homogeneity. Within the Hawaiian radiation, there are two major clades, one of which has retained the ancestral web building behaviour of the genus and comprises ca. 40 species [11]; this lineage shows convergence of web form, but not ecomorphs [15]. The second clade, the “spiny leg” clade, comprises 16 species, and is characterized by abandonment of web building, with the concomitant development of long leg spines and adoption of a vagile, cursorial, predatory strategy [16]. Representatives of the spiny leg clade occur as four distinct ecomorphs associated with specific habitat types: “*green*” on leaves; “*maroon*” on moss, “*large brown*” on tree bark, and “*small brown*” on twigs [7, 11], traits being associated with different feeding behaviours and leg spine morphologies in addition to camouflage [17–19]. Within an ecomorph, different species appear morphologically and ecologically almost identical. Given the exclusively nocturnal behaviour of the spiders and their very limited visual capacity, diurnal predation (presumably by birds) is the most likely selective pressure responsible for the close colour matching [20].

The process of adaptive radiation in the spiny leg *Tetragnatha* is played out across the archipelago, with older islands representing later stages, in which there is generally one representative of each ecomorph of the spiny leg clade in any given native habitat, at least on the older islands. Phylogenetic analyses have shown that the between-island similarity in ecomorphs is largely a result of convergence, meaning that species of one ecomorph can diverge to form a species of a different ecomorph within a given island [7]. To understand the mechanisms involved, we must look to the early stages of the radiation, which are found on the youngest islands of the Big Island and Maui. Moreover, because the youngest island, the Big Island, contains very low diversity, we focus on Maui. Here, there are 7 species of spiny leg *Tetragnatha* representing the four ecomorphs: three species of the *green* ecomorph, *T. waikamoi*, *T. macracantha*, and *T. brevignatha*, one species of maroon, *T. kamakou*, two species of small brown, *T. restricta* and *T. kikokiko*, and one species of large brown, *T. quasimodo*. Importantly, all of these species except for *T. kikokiko* and *T. quasimodo* are contained in a single clade (Fig. 1) [11]; the entire clade is estimated to be approximately 1.4–2.2 Mya [21], similar to the age of the Maui Nui complex, which includes Molokaʻi, Lanaʻi, and Maui.

The specific focus here is on the three species within the *green* ecomorph, and relationships between taxa on the recently (< 15 kyr) connected Maui Nui islands of Lanaʻi (*T. macracantha*) and Maui (*T. brevignatha*, *T. macracantha*, *T. waikamoi*), and the Big Island (*T. brevignatha*) (Fig. 1). Based purely on ecological and morphological data, evidence to date suggests that the different species of the *green* ecomorph appear not to co-occur at a site, with species composition of the three species on East Maui changing markedly between adjacent locations: *T. waikamoi* in Upper Waikamoi, *T. macracantha* in Kīpahulu Valley, and *T. brevignatha* in a mesic area in south west Lower Waikamoi [16]. Populations of each of these species also occur on other volcanoes: *T. waikamoi* on West Maui (Puʻu Kukui), *T. macracantha* on Lanaʻi island, and *T. brevignatha* throughout the Big Island (Kohala, Mauna Kea, Mauna Loa and Hualālai) (Fig. 1). Population genetic studies using mitochondrial, allozyme, and minisatellite markers, have shown that the different populations of each species are highly structured between volcanoes and islands [22, 23]. Therefore, a key question is how the diversification history of these ecologically similar entities has played out in the context of the adaptive radiation, and how this might lead to co-occurrence of different derived ecomorphs. Previous work has suggested that the phenomenon of multiple species on the single volcano may be an “overshoot” effect [7, 24], potentially due to an extinction debt [25]. Yet, how this effect relates to adaptive radiation is unknown.

To address the evolutionary history of the *green* ecomorphs on East Maui, we used a transcriptome-based exon capture approach, which has shown great promise for analysis of population structure for non-model organisms particularly when only very small amounts of DNA are available [26]. We sought to resolve the temporal sequence of events leading to the current distribution of the different species within the *green* ecomorph. In particular, given that species of the *maroon* and *small brown* ecomorph are derived from the *green* ecomorph and largely co-occur with different species of the *green* ecomorph throughout the islands (Fig. 1), can we find evidence of co-occurrence within the same (*green*) ecomorph? This is a critical part of the puzzle in order to understand how divergence between ecomorphs, and subsequent co-occurrence of different ecomorphs, might transpire. If shifts in ecomorph do evolve through competition between closely related species of the same ecomorph [1], at what stage in the process do ecologically similar species interact?

## Methods (Fig. 2)

### Collections

Specimens of *T. macracantha, T. brevignatha* and *T. waikamoi*, all of which display the *green* ecomorph [7], were collected during five field seasons (August 2010, June 2011, January 2012, June 2012 and June 2013; see Additional file 1: Table S1 and Fig. 1). Collecting permits and access were granted by: Department of Land and Natural Resources Hawaiʻi, Division of Forestry and Wildlife Hawaiʻi, the Hawaiʻi Natural Area Reserve System, Hawaiʻi Experimental Tropical Forest, Hawaiʻi Volcanoes National Park, Haleakalā National Park, The Nature Conservancy Hawaiʻi, Kahoma Ranch, Kapāpala Ranch, Kealakekua Ranch, Lanaʻi Resorts LLC, Maui Land and Pineapple, and Parker Ranch. Initial identification was based on morphological attributes of mature animals; however, because the number of mature animals was limited, we also used immature specimens, with their identity assessed from genetic proximity to morphologically identified specimens when identity was ambiguous. Samples were preserved in 95% ethanol at − 20 °C for molecular work. Vouchers have been accessioned in the Essig Museum of Entomology, UC Berkeley (EMEC10201061- EMEC10201174).

**Fig. 2:**
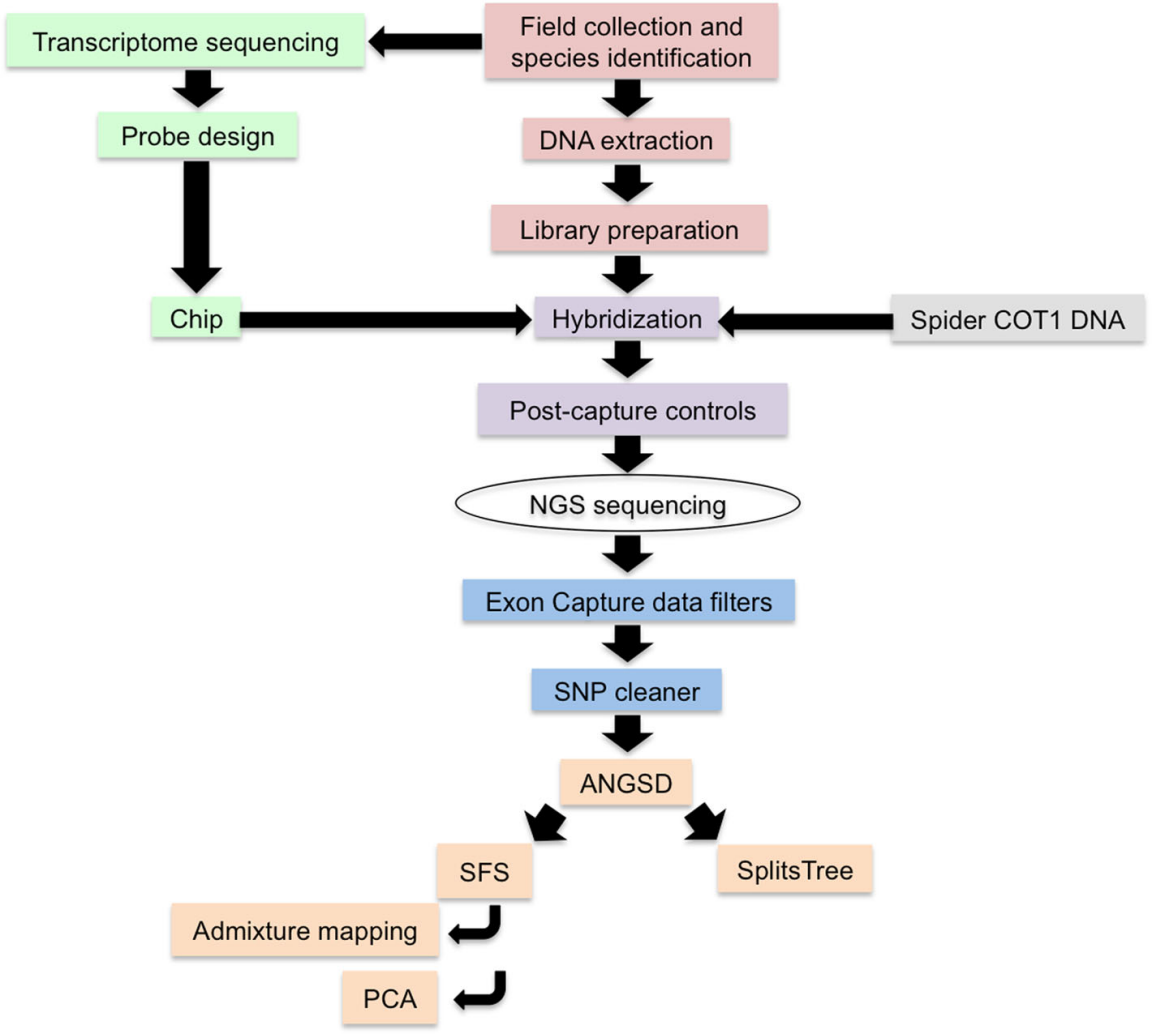
Methodological pipeline before and after NGS sequencing. Before NGS sequencing, the lab work consists of four main elements: (1) probe design (light green), (2) preparation of genomic libraries (red), (3) preparation of spider COT1 DNA (grey) and finally (4) the hybridization experiment (purple). After NGS sequencing there are two main elements: (1) data preparation (blue) and (2) data analysis (orange)

### Transcriptome sequencing and probe design

To obtain a reference transcriptome for the exon capture, we first extracted RNA from a frozen specimen of *T. brevignatha*. The RNA was isolated using a trizol extraction. Library preparation and sequencing was performed by Hudson Alpha (Huntsville, AL. USA). A single RNAseq library was created using the Illumina Truseq RNA v2 kit and sequenced on the Illumina HiSeq2000 aiming at approximately 50 million paired-end reads, 50 bases in length. Probes were designed as outlined in the Additional file 1.

### DNA extraction and library preparation

DNA was extracted from 4 legs of each adult spider, all legs for smaller individuals, and also the cephalothorax for very small individuals, using the Qiagen DNeasy® kit. In very few cases it was necessary to include the abdomen in order to reach the 400–500 ng required for the protocol. Double stranded DNA content was measured using a Qubit® 2.0 Fluorometer (Life Technologies). We performed two replicates for each measurement.

A total of 114 genomic libraries were prepared following the protocol described in [27] (see also Additional file 1). We measured the DNA quality using an agarose/TBE gel and NanoDrop® (2 replicates).

### Exon capture

We performed three hybridization experiments using Agilent SureSelect custom 1 M-feature capture microarrays (see also Additional file 1). To avoid measurement biases due to salt, free nucleotides, primers and/or primer dimers present in the amplified libraries we performed bead cleaning (SeraMag®, General Electric Healthcare Life Sciences) before measuring library concentrations (NanoDrop®). The hybridization procedure followed the protocol of [28] from steps 29 to 61. Post-capture controls are outlined in the Additional file 1.

COT-1 DNA was used to block highly repetitive DNA and to reduce non-specific hybridization, which is important when working with spiders because of the low complexity of their genome with many tandem repeats [29]. The COT1 library (see Additional file 1 for preparation) was used to amplify specific *Tetragnatha* COT1 DNA. For the preparation of *Tetragnatha* COT1 DNA we used a protocol modified from [30], starting with whole body DNA extraction (Qiagen DNeasy® kit) of three large adults of *T. quasimodo*, a species chosen because of its relatively large size among the Hawaiian *Tetragnatha*.

### Hybridization experiment

The effectiveness of the enrichment for the sequences present in the target was verified by measuring shifts towards fewer cycles on the enrichment curve for the positive control using a qPCR. The opposite outcome is expected for the negative control (Additional file 2: Figure S1, Additional file 3: Figure S2 and Additional file 4: Figure S3).

To verify that the fragment distribution after hybridization and whole library amplification was suitable for sequencing we performed a Bioanalyzer® (Agilent Technologies) analysis for each experimental outcome. The three experiments presented a fragment distribution of 200–400 bp, centred around 250 bp (Additional file 5: Figure S4, Additional file 6: Figure S5 and Additional file 7: Figure S6).

### Sequencing platform

The hybridization libraries were sequenced on an Illumina HighSeq 2000 platform (one lane per experiment) with 100 paired-end reads in the Vincent J. Coates Genomics Sequencing Laboratory at UC Berkeley. The raw data is available at the SRA system (https://www.ncbi.nlm.nih.gov/sra) under the accession number SRP065798.

### Exon capture data processing

All scripts used in exon capture data processing are publicly available on the Berkeley Computational Genomics Resource Laboratory (CGRL) github (https://github.com/CGRL-QB3-UCBerkeley/denovoTargetCapturePopGen). They include raw reads filtering, de novo assembly of cleaned data, merging raw assemblies, reconstruction of a reference genome, alignment using Novoalign (http://www.novocraft.com), duplicate removal, and coverage estimations. For more details on analytical pipeline see Additional file 1 [31, 32].

### Raw SNP filtering and variant calling using ANGSD

Given the low coverage of our data we used Analysis of Next Generation Sequencing Data (ANGSD) [33] to calculate genotype likelihoods rather than genotype calling for most of the downstream analyses. Before using ANGSD we selected the sites that will be used in the analysis. The first step was to use SAMtools [34] to produce some of the data quality control information in VCF format, which were then further filtered using SNPcleaner [35]. Several site-specific filters implemented in this program were applied [31]. The resulting sites that passed all initial filters were passed to ANGSD for SNP calling and estimating allele frequencies for each population. Detailed methodologies of ANGSD can be found in [33].

To use the program SplitsTree (see below), we performed variant calling using ANGSD to generate the respective input files. For this we took the cleaned the Forward, Reverse and Unpaired files for each specimen and mapped them against the same reference (*T. brevignatha*). The new bam files were subsequently used to re-run ANGSD, using extra filters (-doGeno 2 -SNP_pval 0.1 –postCutoff 0.75 -geno_minDepth 3 -minInd 81 -doCounts 1 -doGlf 4). These filters were applied in order to obtain high quality data and remove sites with lower coverage (−geno_minDepth) than a determined cutoff (-minInd). We chose to keep sites with a minimum of 3× in at least 70% of the individuals. The resulting genotype file was then converted into a nexus file for Splitstree V4.13.1.

### Population genomic analyses

#### Principal component analysis (PCA)

To assign juveniles and to determine the relative clustering of all the specimens studied, we performed a PCA using the program ngsCovar that is implemented in ngsTools [35] package. For more details about the generation on the PCA input data see Additional file 1.

#### Admixture analysis

To use NgsAdmix we followed the commands present on the web site of the program (http://www.popgen.dk/software/index.php/NgsAdmix). All the specimens were mapped against the same reference (*T. brevignatha*). The generated bam files (duplicates removed) were then used to produce Beagle files [36] using ANGSD.

For each value of K, we performed 10 replications and plotted the run with the highest likelihood value that was repeated (Skotte *pers. comm.*). We tested the grouping of the specimens with K values from 1 to 10. For each value of K, the average likelihood value was plotted and the reduction in the slope was used as a reference for the number of population partitions (Additional file 8: Figure S7) [37].

#### Phylogenetic network analysis

To investigate phylogenetic relationships between different populations of the three closely related species, we constructed an unrooted phylogenetic network using the default parameters in the program SplitsTree V4.13.1 [38]. This approach allows a collection of incompatible trees, equally consistent with a given data set, to be represented [39]. This situation is common in cases of hybridization, horizontal gene transfer, recombination, and gene formation/duplication/loss. In particular, due to the known role of admixture in early speciation events [40, 41], this method is particularly suitable for our system.

#### Genetic differentiation (F_ST_)

The first step was to run ANGSD for all the populations of interest. We merged the files that contained all the used sites (*.pos) and selected only the unique sites (intersect.keep). Then, we used the option FST in the program PopGenTools_2.76.pl in order to run multiple comparisons across different populations (*T. brevignatha*, Maui; *T. brevignatha*, Big Island; *T. macracantha*; *T. waikamoi*) and between the two populations of *T. waikamoi* (East and West Maui) and *T. macracantha* (Lanaʻi and East Maui).

*Neutrality test (Tajima’s D)*: We first estimated the folded SFS using ANGSD and then generated a theta file for each population. As part of the options we had to include <−GL 1 –fold 1 –anc>. The “ancestral genome” (−anc) corresponded to the same file as the “reference” (−ref). ANGSD outputs a file with Tajima’s D values for each contig.

To obtain an exome-wide estimation of Tajima’s D we created a new theta file where all the sites were present in the same chromosome. Then, we used the thetaStat program to estimate Tajima’s D [42].

*Nucleotide diversity (π)*: From the exome-wide Tajima’s D output file *.thetas.gz.pestPG we extracted the value of theta P (tP) and divided by the number of sites used for that population.

## Results

### Transcriptome-based exon capture

Transcriptome sequencing of *T. brevignatha* yielded 14,664,987 bp raw read data. The final target size of the capture consisted of 1.7 Mb which corresponds to 1826 inferred ORFs from the whole transcriptome sequencing. With a probe length of 60 bp and a tailing density of 2 bp we generated a final set of 967,487 probes, made up of 814,187 original probes plus 153,300 added to the terminal ends of each ORF. Extra probes were added to the ends of each ORF to increase the coverage [26]. 114 genomic libraries were successfully prepared and indexed. A basic evaluation of exon capture performance of the three experiments is available in the Additional file 1.

### Data analyses

#### Principal component analysis (PCA)

**Fig. 3:**
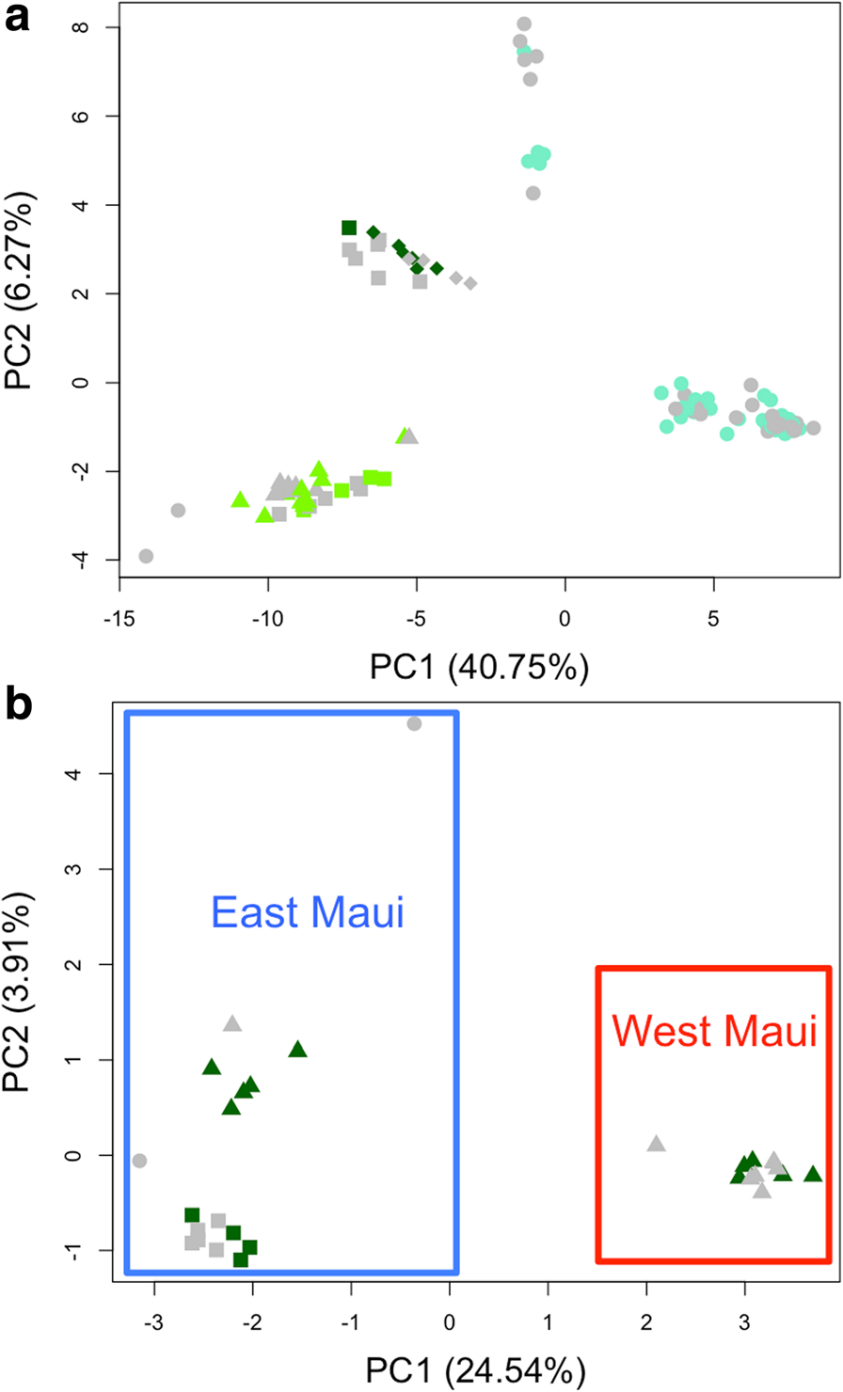
Principal Component Analysis of (**a**) PC1 vs PC2 of all the specimens combined and (**b**) PC1 vs PC2 of the specimens clustered with *T. waikamoi.* The shapes represent the localities as indicated on Fig. 1. The colours represent the species identification based on morphology. Capypso: *T. brevignatha*; Dark green: *T, macracantha*; Light green: *T. waikamoi* and Grey: juveniles or specimens that could not be identified with certainty. The two groups of *T. brevignatha* indicate the Maui and Big Island populations

To assess the number of genetically distinct population clusters we performed a Principal Component Analysis. The first two Principal Components (PC1: 40.70% and PC2: 6.61%) show four well-defined groups (Fig. 3a), as do PC1 (40.70%) and PC3 (5.65%) (Additional file 9: Figure S8). *T. brevignatha* is split into two groups: Maui and Big Island. The third recognizable group includes most of the *T. macracantha* specimens, with populations from Lanaʻi, Koʻolau and the three specimens from Kīpahulu Valley that cluster with Koʻolau. The fourth group corresponds to *T. waikamoi*, including specimens from Kīpahulu valley and Lower Waikamoi, sites from which only *T. macracantha* and *T. brevignatha*, respectively, have been reported.

To gain more detailed insights into the distribution of *T. waikamoi* we performed a PCA with only those samples. The first two PC’s (PC1: 24.54% and PC2: 3.91%) (Fig. 3b) show a clear separation of the two volcanoes (East and West Maui). On East Maui, the Upper Waikamoi population shows little differentiation along PC1 from the Kīpahulu population. One of the two specimens from Lower Waikamoi lies between these two groups, while the second is separated from the rest, but mostly along the PC2 (3.91%).

#### Admixture analysis

To investigate the presence of hybridization we used the program NgsAdmix [43] for estimating admixture proportions of low coverage sequencing data using genotype likelihoods. NgsAdmix was not developed to identify an optimal number of clusters (Skotte *pers. comm*., Crawford and Nielsen *pers. comm.*, for a general discussion [44]). Thus, while the inability of independent runs to reach convergence indicates that K values are too high or too low, it is not possible to select among K values where there are consistent convergences. For our data, the values of K converged for independent runs between K = 2–5. For the current study, the preferred grouping was K = 5. When grouping the specimens into only two groups (K = 2; Additional file 10: Figure S11a) the populations of *T. macracantha* (Kīpahulu Valley, Koʻolau and Lanaʻi) and *T. waikamoi* (Kīpahulu Valley, Upper Waikamoi and Puʻu Kukui) are grouped together (blue). The other group (green) corresponds to the Big Island populations of *T. brevignatha*. All individuals of the Maui population of *T. brevignatha* (Lower Waikamoi; LWKM) show equal admixture proportions, which could indicate hybridization. However, the effect is most likely an artefact of the K value being too small to allow for differentiation of this group (see K = 3 below). Two specimens collected in Lower Waikamoi correspond to *T. waikamoi* (Additional file 10: Figure S11a).

**Fig. 4:**
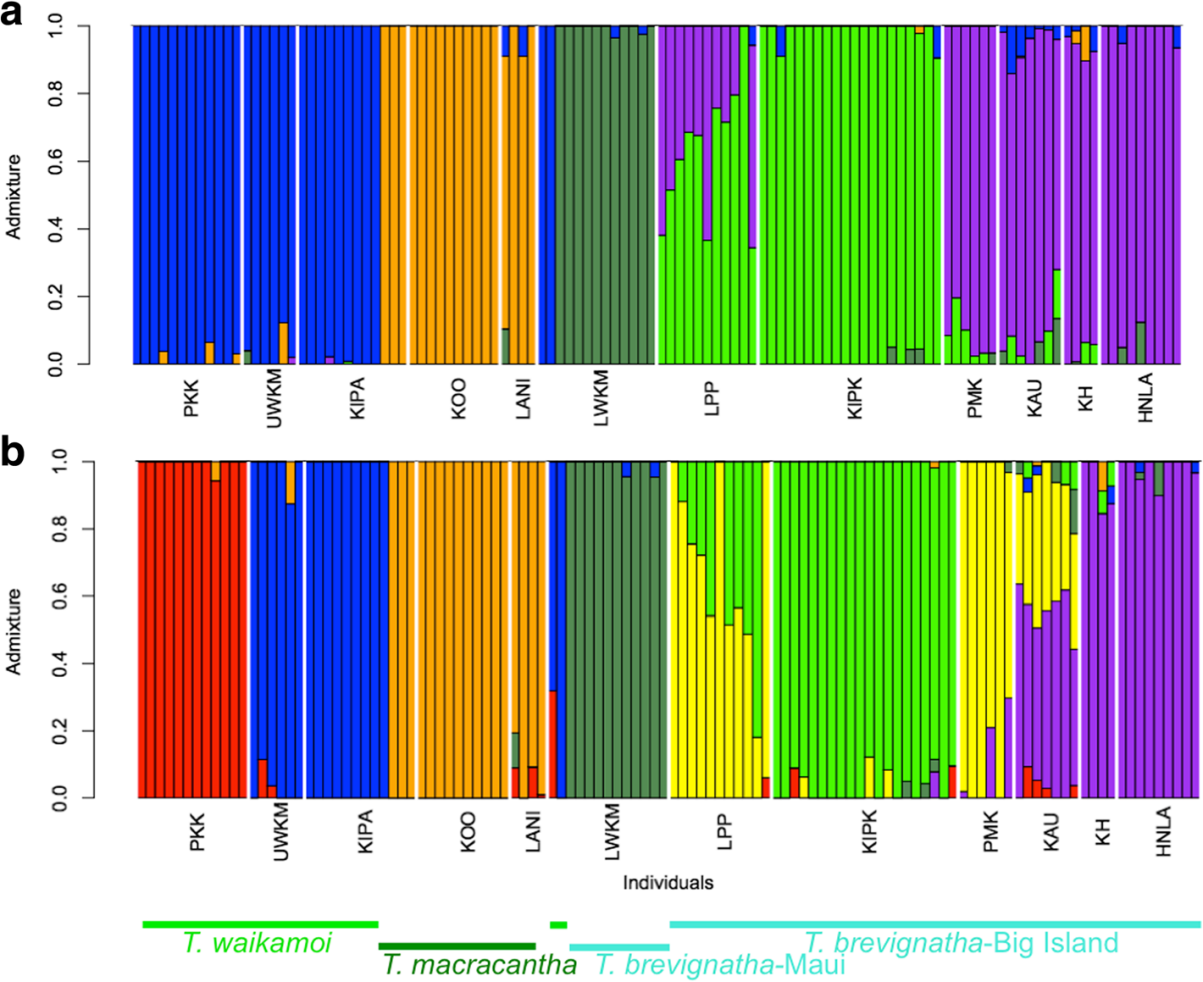
Population structures determined by NgsAdmix runs for **a** K = 5, and **b** K = 7. The specimens are separated by localities. The localities are: PKK (Puʻu Kukui), UWKM (Upper Waikamoi), KIPA (Kīpahulu Valley), KOO (Koʻolau), LANI (Lanaʻi), LWKM (Lower Waikamoi), LPP (Laupāhoehoe), KIPK (Kīpuka), PMK (Puʻu Makaʻala), KAU (Kaʻu), KH (Kona Hema) and HNLA (Honoaula). See Fig. 1 for geographic reference. Specimens of each species are indicated by the horizontal bars at the bottom of the figure. Capypso: *T. brevignatha*; Dark green: *T, macracantha*; Light green: *T. waikamoi.* Note, the two *T. waikamoi* individuals in LWKM

For K = 3 (Additional file 11: Figure S12b) most of the specimens from the Maui population (UWKM) of *T. brevignatha* form a new group. It is also possible to identify low levels of admixture (< 20%) in some specimens. When 4 ancestral populations are selected (K = 4) (Additional file 11: Figure S12c), the group consisting of *T. macracantha* is recovered (orange). For K = 5 (Fig. 4a), the Big Island populations of *T. brevignatha* are separated into two groups: Windward (Laupāhoehoe and Kīpuka) and Leeward+South (Puʻu Makaʻala, Kaʻu, Kona Hema and Honoaula). The separation between the East Maui (Upper Waikamoi) and West Maui (Puʻu Kukui) populations for *T. waikamoi* is only seen at K = 7 (Fig. 3b).

#### Phylogenetic network analysis

**Fig. 5:**
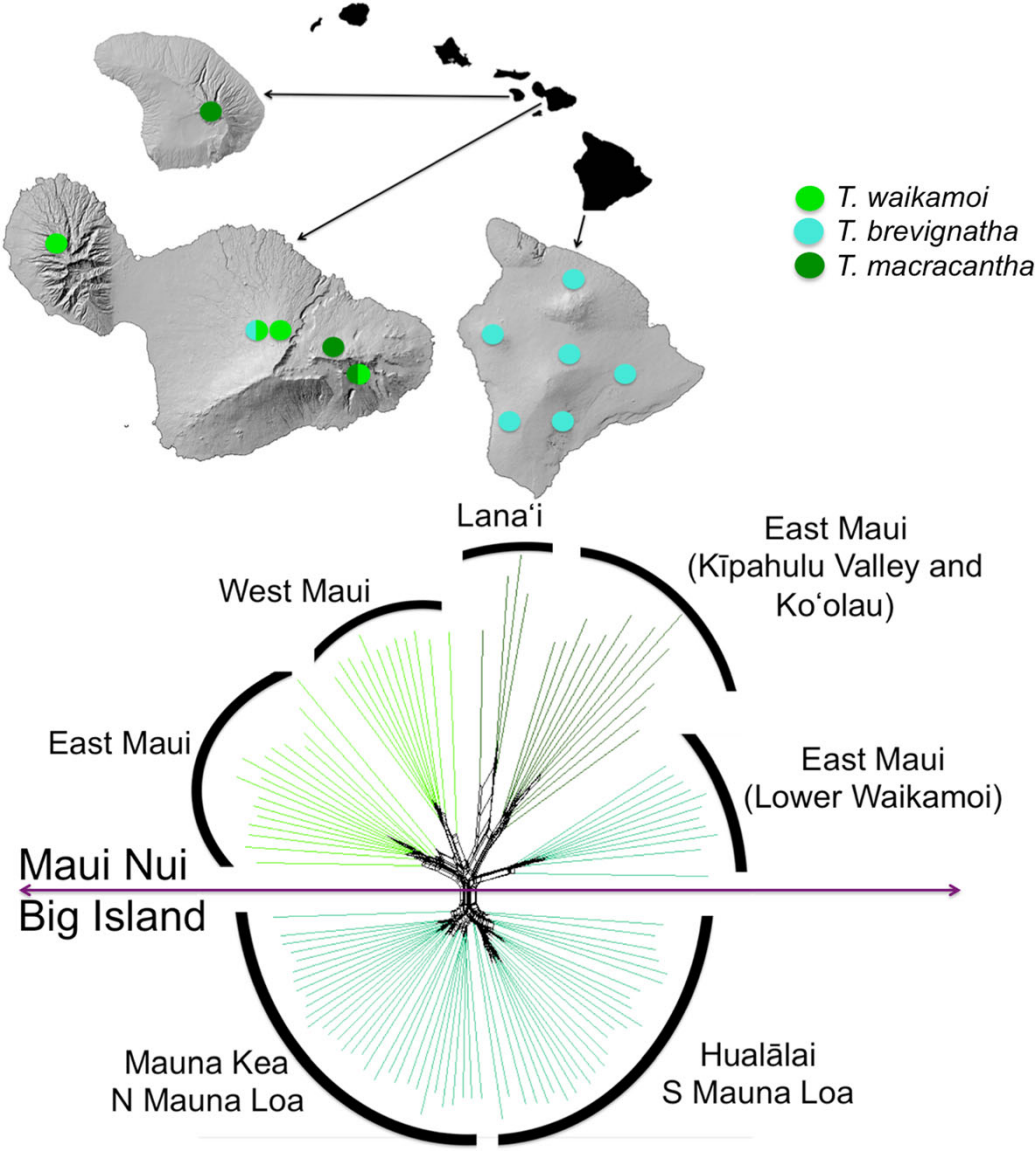
Phylogenetic network of all specimens and populations of the three species of the green ecomorph. The network was created with SplitsTree V4.13.1. The color code is the same as in Figs. 1 and 3. The labels indicate the localities where those specimens were collected, also illustrated in the map. Note that in two localities on East Maui where *T. waikamoi* specimens are found in sympatry with another species of the same ecomorph. http://www.hear.org/starr/maps/stock/

To analyse the relationships between species in a phylogenetic framework and represent the potentially shared SNPs between them, we generated a phylogenetic network. The phylogenetic relationships among the different populations are shown in Fig. 5. All the sequenced specimens were included and a total of 8397 sites were used. The significantly smaller number of sites used compared to the other low coverage applications was due to the filters applied for this particular application (see Materials and Methods). The phylogenetic network consists of 359 split events and shows 5 clearly distinct groups: East Maui; West Maui; Lanaʻi + East Maui (Kīpahulu Valley and Koʻolau); East Maui (Lower Waikamoi), and Big Island (Mauna Kea, N Mauna Loa, Hualālai and SW Mauna Loa) (Fig. 5). The first two groups correspond to two populations of *T. waikamoi*; the third is *T. macracantha*; and the last two are Maui and Big Island lineages of *T. brevignatha*, respectively. Note that when looking more closely, the group Lanaʻi + East Maui (Kīpahulu Valley and Koʻolau) can be divided in two subunits: Lanaʻi and East Maui (Kīpahulu Valley and Koʻolau). This separation was not evident on the PCA or admixture plots. In a similar way, the Big Island can be divided in two large fractions; on one side Mauna Kea and N Mauna Loa, and on the other Hualālai and S Mauna Loa.

The Maui specimens of *T. brevignatha* were all collected at the same site within which there is no clade structure, though this population is very distinct with respect to the Big Island. The other large group consists of specimens of *T. macracantha*. Within this group there is a very clear break between the specimens from Maui (Koʻolau and a few from Kīpahulu Valley) and Lanaʻi. The fourth well-defined group includes all the specimens from the West Maui (Puʻu Kukui) population of *T. waikamoi*. Finally, the East Maui population of *T. waikamoi* includes specimens from multiple sites on East Maui [16]. This clade shares a common stem with the West Maui (Puʻu Kukui) population of *T. waikamoi*.

#### Genetic differentiation (F_ST_), neutrality test (Tajima’s D) and nucleotide diversity (π) (Additional file 1: Tables S3 and S4)

The patterns previously described were quantified using F_ST_, Tajima’s D, and π in a complementary manner. First, the nucleotide diversity of *T. macracantha* (0.0042) and *T. waikamoi* (0.0040) was higher than *T. brevignatha* from Maui (0.0025) and *T. brevignatha* from Big Island (0.0022).

Within *T. macracantha*, the Maui population shows almost double (0.0041) the nucleotide diversity of the Lanaʻi population (0.0028). Although the sample size from Lanaʻi was small, this difference could be interpreted as the population from Lanaʻi being derived from East Maui, with the small Lanaʻi population affected by drift. The *F*_*ST*_ between both populations is 0.24. On the other hand, the West Maui (0.0026) and East Maui (0.0028) populations of *T. waikamoi* have similar values of nucleotide diversity making it difficult to assess which one was the first to be established. The *F*_*ST*_ between East and West Maui is 0.30. These *F*_*ST*_ values are two times lower than the estimated Global *F*_*ST*_ (0.66). This could also indicate a rather recent split of the two populations. For *T. brevignatha,* the *F*_*ST*_ value between populations on different islands (*F*_*ST*_ = 0.74) is entirely consistent with previous work [22].

We found negative Tajima’s D values for all investigated populations (Additional file 1: Table S3; Additional file 12: Figure S12), which could indicate population growth after a recent bottleneck. The largest difference is between *T. brevignatha*, Maui (− 1.10) and *T. brevignatha*, Big Island (− 2.29), with more than a 2-fold increase. The same tendency is observed when plotting the contig Tajima’s D (Additional file 12: Figure S12). However, values smaller than − 2 and bigger than 2 are usually not significant.

## Discussion

Our study sought to examine genetic events that characterize the early, non adaptive, stages within an adaptive radiation, asking specifically whether closely related species of the same ecomorph of Hawaiian spiny leg spiders co-occur, and if so whether they exclude each other, introgress or admix, or simply contribute to the overall genetic diversity of populations. To address this question, we used a transcriptome-based exon capture approach that is well suited for addressing questions of complex population structure over recent time scales.

The most striking result of our study is that there are areas on East Maui where species that share the same ecomorph co-occur. More precisely, *T. waikamoi* occurs across East Maui, and can be found in sympatry with either *T. macracantha* or *T. brevignatha*. While it might be surprising that apparent ecological equivalents can co-occur, it is clear that co-occurrence (1) may follow a period of allopatry [21] and (2) is uncommon and never found on any of the older volcanoes [7], so presumably is short-lived during the process of adaptive radiation, and limited to a few sites on East Maui. Importantly, this is the period during the course of adaptive radiation at which ecological equivalents co-occur, which presumably then sets the stage for competition leading to ecological divergence and the formation of different ecomorphs. Finding multiple species of the same (*green*) ecomorph together provides the missing link between early non-adaptive divergence within the same ecomorph in allopatry and later co-occurrence of different ecomorphs.

Co-occurrence of similar ecological forms in adaptive radiations is seldom observed. In situations where it has been documented, often the species are not as close ecologically similar as originally thought [45]. For example, in cichlid fish, similar ecomorphs often co-exist in Lake Tanganyika [46]. Yet in this case, the similar ecomorphs are not closely related species and the proposed explanation is that the older age of the adaptive radiation of cichlids in Lake Tanganyika allows niche separation of similar ecomorphs due to small differences that have accumulated over time. Similarly, in the adaptive radiation of *Anolis* lizards in the Caribbean, co-occurring species of the same ecomorph appear to have differentiated based on their body size [47] or according to thermal preference [48]. The co-occurrence of multiple species of trunk-ground ecomorphs on Cuba in particular is thought to be due to secondary sympatry, with divergence in thermal preference allowing coexistence. However, many questions remain regarding the role of thermal resource partitioning in allowing species coexistence [49], and, for the *Anolis*, the island of Cuba provides only a single, and quite ancient, snapshot in time, making it difficult to infer process. The important insight gained from the current study is that we can use the ages of the islands to time the sequence of events during early stages of adaptive radiation. Thus, in contrast to the other systems, co-occurrence of species of the same ecomorph appears to be limited to a short period prior to ecological divergence.

A second important finding of the current study is the lack of evidence of ongoing genetic admixture between these closely related species found in sympatry. This marked genetic segregation of populations is particularly striking given that *T. waikamoi* can occur in sympatry with the other two *green* species from East Maui (Fig. 5). Species-specific PCAs for *T. macracantha* and *T. brevignatha* including the sympatric population of *T. waikamoi*, show clear genetic segregation between each species. In the case of *T. macracantha* (Additional file 13: Figure S9), the distinction from co-occurring specimens of *T. waikamoi* occurs along PC1 (22.93% of the variation). Individuals of *T. waikamoi* that co-occur with *T. brevignatha* are clearly separated along PC2 (Additional file 11: Figure S10). In neither case is there evidence of admixture. The admixture analysis (Fig. 4) also supports the lack of ongoing hybridization among co-occurring species: While for K = 2 there is a suggestion of historic hybridization at one of the localities (Lower Waikamoi; LWKM) where *T. waikamoi* and *T. brevignatha* co-occur (Additional file 10: Figure S11a), the equal admix proportions between the populations of these two species suggests that hybridization is not ongoing. All the individuals from Lower Waikamoi differentiate into a distinct population at K = 3 (Additional file 10: Figure S11b). Then, for K = 4, the other locality with 2 species (Kipahulu Valley, KIPA) shows the separation between *T. waikamoi* and *T. macracantha* (Additional file 10: Figure S11c). The lack of admixture is also indicated by the well-defined groups present in the phylogenetic network (Fig. 5). These observations suggest that strong reproductive barriers have already been built between ecologically similar species. Such barriers might be instrumental for allowing the transitory peak in diversity [13, 25], generated by the co-existence of different genetic lineages with similar ecologies [7]. Studying the nature of these barriers (pre or post zygotic) is one of the next steps to understand the mechanistic basis of the early stages of community assembly in this adaptive radiation.

A third major finding of the current study is the possibility of multiple colonisations between the youngest islands. Historical reconstruction of such complex events is not straightforward. However, considering the data generated in the current study, together with a somewhat broader phylogenetic context [21], the simplest (though certainly not the only) scenario is that a common ancestor of the Maui Nui clade was originally established throughout Maui, Lanaʻi; and then the Big Island. Taxa subsequently differentiated allopatrically on the different volcanoes. The existence of multiple lineages on East Maui might then be the result of secondary dispersal of the differentiated taxa on to this single landmass. This would suggest that all speciation events were on different islands or volcanoes, with current distributions due to secondary colonization.

An important implication of these results is that these early lineages have dispersed widely, suggesting that the *green* ecomorph species are not inherently dispersal limited; rather, limits to dispersal may be due to exclusion. A similar inference has been derived for the case of Hawaiian *Laupala* crickets, where there is evidence for multiple back-migrations between the youngest islands [50]. Likewise, genetic data reveal considerable interisland movement among weevils from the Canary and Madeira Islands [41]. Unlike the spiders, the outcome of the multiple colonisations among both crickets and beetles appears to be admixture. However, recent phylogeographic studies of Hawaiian planthoppers are also starting to reveal patterns of higher than expected movement, at least between the younger islands, and without evidence of admixture (Goodman et al. unpublished data). Thus, the data are increasingly suggesting that interisland migrations are not uncommon between the youngest islands [10].

The observation of a large number of genetic lineages on East Maui, is also consistent with concept of extinction debt [25]. This idea postulates that, as species accumulate over evolutionary time, there may often be a considerable lag between the species diversity and the carrying capacity of the area, as demonstrated recently for islands [51], including the Hawaiian Islands [13, 25]. Indeed, depending on the lineage, such diversification might happen so slowly within an archipelago that the lineage may never reach its carrying capacity. Alternatively, species numbers may reach carrying capacity quite quickly, with the extinction debt carried over for extended periods. Following these arguments, the multiple species and genetic lineages of the same ecomorph on East Maui may well represent extinction debt, with older volcanoes and islands all showing the results of ecological and evolutionary processes leading to a single representative of a given ecomorph [7].

## Conclusion

The current study examined species diversification across the recent stages of a geological chronology to provide insights into the early stages of adaptive radiation: Specifically, if we assume that the early stages of adaptive radiation involve allopatry of taxa within the same ecomorph, how do we get to the next step of co-occurrence of derived taxa across different ecomorphs? We assessed evidence for co-occurrence within the same ecomorph at the early stages of adaptive radiation, and the possible outcomes of co-occurrence events, i.e., competitive exclusion, hybridization and genetic admixture, or simply haphazard accumulation of genetic diversity within the ecomorph. The results show that species within the same ecomorph can, and do, co-occur in the early stages of the adaptive radiation process. However, the very brief period within which one finds co-occurrence of taxa within the same ecomorph, suggests that competitive exclusion plays a major role. Yet, this short period of co-occurrence of closely related species allows the necessary interaction predicted for competition leading to ecological specialization [1]. Moreover, there is little evidence for hybridization at this stage: Although mixing may have been important in creating diversity in the closely related species leading to this point, these species are now distinct genetic entities. The results highlight the dynamic nature of young communities in the early stages of a well-studied adaptive radiation and provide a possible explanation for a pattern early in the process of diversification whereby species diversity can be higher than expected [7, 12, 50]. It is also consistent with an apparently transitory peak in diversity that has been found in multiple lineages across several archipelagoes [51]. Thus, our results support the hypothesis that the early stages of adaptive radiation are characterized by accumulation of ecologically similar taxa, giving way to the sorting and divergence of ecological forms over evolutionary timescales [10].

## Additional files

Additional file 1: Supporting Information. (DOCX 183 kb)

Additional file 2: **Figure S1.** qPCR controls Experiment 1. (TIFF 4517 kb)

Additional file 3: **Figure S2.** qPCR controls Experiment 2. (TIFF 5307 kb)

Additional file 4: **Figure S3.** qPCR controls Experiment 3. (TIFF 3510 kb)

Additional file 5: **Figure S4.**Bioanalyzer read of the fragment distribution of the whole library after amplification (Experiment 1). (TIFF 3975 kb)

Additional file 6: **Figure S5.** Bioanalyzer read of the fragment distribution of the whole library after amplification (Experiment 2). (TIFF 5364 kb)

Additional file 7: **Figure S6.** Bioanalyzer read of the fragment of the whole library after amplification (Experiment 3). (TIFF 5162 kb)

Additional file 8: **Figure S7.** Average Likelihood (10 independent replicates) vs K. (TIFF 4748 kb)

Additional file 9: **Figure S8.** Principal Component Analysis of all the specimens. a PC1 vs PC3 and b PC2 vs PC3. (TIFF 3782 kb)

Additional file 10: **Figure S11.** NgsAdmix runs. a K=2, b K=3, c K=4, d K=6, e K=8, f K=9 and g K=10. (TIFF 5897 kb)

Additional file 11: **Figure S10.** Principal Component Analysis of T. brevignatha and its sympatric population of T. waikamoi (Lower Waikamoi). (PNG 114 kb)

Additional file 12: **Figure S12.** Gene by gene Tajima’s D. (TIFF 9982 kb)

Additional file 13: **Figure S9.** Principal Component Analysis of T. macracantha and its sympatric population of T. waikamoi (Kipahulu Valley). (TIFF 6195 kb)
