# Supplementary material for: Co-occurrence of ecologically similar species of Hawaiian spiders reveals critical early phase of adaptive radiation
Source: BMC Evol Biol. 2018 Jun 19;18:100. doi: 10.1186/s12862-018-1209-y (PMC6009049; doi:10.1186/s12862-018-1209-y)
Supplement: Supplementary file 1 — Supporting Information. (DOCX 183 kb) [file 12862_2018_1209_MOESM1_ESM.docx]

**Additional file 1: Supporting Information**

**Probe Design**

The Probe design used a modified version of the pipeline and the scripts deposited in the Computational Genomics Resource Laboratory (CGRL)-QB3-UCBerkeley Github site (https://github.com/CGRL-QB3-UCBerkeley). Detailed instructions for each script can be found on that site. We followed the steps described below:

1.- *Quality control and sequence assembly:* The raw reads were trimmed based on per-read quality scores, primer artifacts were removed, and reads were filtered for length using the FASTX Toolkit (Hannon, [hannonlab.cshl.edu/fastx_toolkit/‎](http://hannonlab.cshl.edu/fastx_toolkit/%E2%80%8E)).  All sites in a read occurring after a quality score of 20 or less were removed and all reads less than 30 bp in length afterwards were deleted.  The first nine bases of each read were removed to eliminate primer/sequencing artifacts.  The quality of processed reads was assessed using FASTQC (FastQC, [bioinformatics.babraham.ac.uk/projects/fastqc/‎](http://bioinformatics.babraham.ac.uk/projects/fastqc/%E2%80%8E)).  The individually processed paired-end read files were resynchronized using a custom script. Each transcriptome was assembled using the Trinity pipeline [1] requiring a minimum contig length of 100 bases and minimum kmer coverage of 2.

2.- *Contaminant removal:* The resulting contig files were filtered for mitochondrial (BLASTX), ribosomal (BLASTN), and contaminant sequences as determined by BLASTX and MEGAN4 analyses of the deeply sequenced *T. grallator* somatic transcriptome [2] (BLASTN) using custom BLAST databases and searched (BLASTX) against the NCBI non-redundant (nr) protein database.

3.- *Visualization of BLASTx result*: Using MEGAN [3] we classified each read into the lowest taxonomic unit. For some genes there is good evidence that they belong to a specific low taxonomic unit, so they were included in that group. Others were classified into a more generic node, because there is no evidence that they go into lowest levels. We were as stringent as we could in order to not add contaminants. All the sequences with hits to non-metazoan or nematodes were removed.

4.- *ORF prediction:* Putative open reading frames (ORFs) were predicted from the contigs remaining after contaminant removal using Trinity’s transcripts_to_best_scoring_ORFs.pl script, minimum length 50 amino acids.

5.- *Transcriptome coverage estimation:* It was assessed by comparing each assembly to a standard set of 248 core eukaryotic genes (CEGs) using the program Cegma [4].  These CEGs were derived from the eukaryotic orthologous groups (KOGs), a subset of the Cluster of Orthologous Groups (COGs) of Proteins database [5].

6.- *Select gene families (“comps” is the denomination of gene families from TRINITY) with single representative in order to avoid paralogs*: The following line of BASH code generates a list without sequences. It uses as an input the result of TRANSDECODER.

>grep "^>" Name_nrBLAST_out-ex.fasta.transdecoder.cds | cut -d"_" -f1 | sort | uniq -u > Name_cds_uniq_comps.txt

7.- *Change line break by an underscore*: This is necessary for the downstream analysis.

>sed 's/$/_/g' Name_cds_uniq_comps.txt > Name_cds_uniq_comps2.txt

8.- *Estimate the number of ORFs:* We used the following BASH command:

>wc -l Name_cds_uniq_comps2.txt

9.- *Create FASTA file with no paralogs*: Search in the fasta file (<Name_nrBLAST_out-ex.fasta.transdecoder.cds>) for all the sequences that are singleton families (<Name_cds_uniq_comps.txt>; this is the output file of step 6). This will create a file that has all the information of the sequences present in the list (<Name_cds_NoParalogs.fasta>).

>cat Name_cds_uniq_comps.txt | while read line; do grep -A 1 $line Name_nrBLAST_out-ex.fasta.transdecoder.cds_nent.fasta; done > Name_cds_NoParalogs.fasta

10.- *GC filer:* This filter takes out sequences with %GC below 30 and above 70. This program is available upon request and it is not in the CGRL Github site. Script used: GC_percentage_calc.py

11.- *kmer count:* The kmers were counted using the JELLYFISH program implemented in TRINITY [1]. We used a kmer size of 15 (-m 15)

12.- *Write the tab delimited kmer count file:* We created that file using the JELLYFISH program implemented in TRINITY [1].

13.- *Indexed file*: This file is created using the program: indexer_15mers.pl.

14.- *Symbol changes*: For downstream analysis we did some notation changes, which make more convenient the use of the different files.

>sed 's/>/>chr_/g' Name_cds_NoParalogs_goodGC.fasta | sed s'/ /_/g' > Name_cds_NoParalogs_goodGC_4ProbeDesign.fasta

15.- *Create the target region file*: It uses as an input the file <Name_cds_NoParalogs_goodGC_4ProbeDesign.fasta> in the program target_file_maker.py, which is available upon request.

16.- *Array design*. Using the script array_design_modified.pl the probes were design. We used a probe length of 60 bp (-p 60) and a tailing density of 2 bp (-t 2). We did not include any flanking region (-f 0), because for spiders the Intron-Exon boundaries are not known. For the same reason the tailing density was so high.

17.- Preparation of the array by Agilent (Santa Clara, CA. USA).

Another important thing to consider during the array design is to remove mitochondrial genome to avoid over representation during the capture experiment. The mitochondrial genome has so many copies and their sequences could completely out compete other sequences to capture. Only one mitochondrial gene is included in the chip in order to have a control to test for empirical error rates if needed. Alternatively a sex-linked gene could be used for this purpose. The only caveat is that it will only be possible to use on the samples where this sex chromosome is present. In general as a way to check for empirical error rate, it is necessary to use a haploid marker.

**COT 1 DNA library preparation**

We started with 13 μg. of high molecular weight DNA. The DNA quality was verified on an agarose/TBE gel and the amount calculated based on a concentration estimation measured with Qubit® 2.0 Fluorometer (Life Technologies).

The DNA was fragmented using a Bioruptor® Standard (Diagenode) set in the “High” mode and following a fragmentation cycle of 3.5 minutes on, change half of the cold water and then 3.5 minutes on again. It was repeated 3 times or until reach a fragment distribution between 100 bp and 500 bp, centered around 250 bp. The fragment distribution was checked on an agarose/TBE gel. After fragmentation the remaining amount was 4.31 μg. Then, we did an ethanol precipitation to purify the DNA.

The DNA denaturation was done for 5 to 10 minutes at 95°C. Then, 12 volumes of SSC were added and the reannealing was done at 60°C for 22 minutes. This amount of time was calculated based on the formula described in [6]. Immediately after the 22 minutes we proceed to the S1 nuclease (Thermo Scientific) hydrolysis, which removed the single stranded DNA. All the reagents involved in this enzymatic digestion and free nucleotides were removed with another ethanol precipitation. After the S1 nuclease treatment it was 1.031 μg, which corresponds to the 23.9% of the original amount. For humans and other vertebrates the percentage of COT1 DNA present in the genome is around 10% (Singhal *pers. comm.*). However, spiders have many repetitive elements and low complexity DNA [7, 8]. For this reason it is not as surprising that the amount of COT1 DNA is relatively high.

The isolated COT1 DNA is immortalized by a regular library preparation for NGS [9]. We used custom designed COT1 adaptors (cot-1 Forward adapter: AGCTATCAGACTCGGACTACTGATGCAGTG; cot-1 Reverse adapter: CACTGCATCAGT). For the indexing PCR step we used the same primer for the reverse and forward direction (cot 1 primer: AGCTATCAGACTCGGACTACTG). These sequences were later removed by an infrequent restriction enzyme (BstI) (New England Bio Labs), which has a restriction site in the COT1 DNA primers. We used 2 units (2U) of enzyme per each μg of COT1 DNA. After this enzymatic digestion we proceed to clean the product by a beads clean up protocol as described in [9].

A final amount of 50 μg of COT1 DNA was required for each hybridization experiment (3 in total). More than 100 PCR reactions were required to reach this quantity. Note, that the PCR product had to be purified with a beads clean up before use it. We did 15 cycles of the indexing PCR protocol described in [9]. The amount of template that we found optimizes the amount of PCR product and how much of the original COT1 DNA library we were able to use per reaction was 20 ng.

Higher numbers of cycles lead to problems due to the formation of secondary structures and daisy chaining. These high molecular weight structures were not digested by the restriction enzyme, which removed the indexing primers. Initially we also tried using PCR product as a template for a new PCR reaction, however it was found not to be a good approach because after 2 or 3 iterations there was a substantial accumulation of high molecular weight DNA.

The final 50 μg of COT 1 DNA had to be concentrated on a SpeedVac (CentriVap DNA Concentrator, LABCONCO) in order to reach the volume required in the hybridization experiment [10].

**Genomic library preparation**

A total of 114 libraries were prepared following the protocol described in [9]. The details of the localities are in Table S1. The DNA quality was verified on an agarose/TBE gel and the amount calculated based on a concentration estimation measured with Qubit® 2.0 Fluorometer (Life Technologies) (2 replicates). The amount of starting material ranged from 400 to 500 ng depending on the sample.

The DNA was fragmented using a Bioruptor® Standard (Diagenode) set in the “High” mode following a fragmentation cycle of 3.5 minutes on, change half of the cold water and then 3.5 minutes on again. It was repeated 3 times or until it reached a fragment distribution between 100 bp and 500 bp, centered around 250 bp. The fragment distribution was checked on an agarose/TBE gel. The first 50 samples were prepared using cold water and ice in the Bioruptor® Standard. For the following 64 we used a water cooler system (Diagenode). The fragmentation protocol remained the same. Right after fragmentation we performed a beads clean up in order to concentrate the samples and eliminate the small fragments of DNA.

The only difference with respect to the original protocol is that we used Sera-Mag magnetic beads (GE Healthcare Life Sciences) instead of AMPure® XP beads (Agencourt). However, it does not affect the result of the protocol (Smith *pers. comm.*). For every batch of libraries prepared we used a 150 bp positive control (fragment of H3) to check the success of the adaptor ligation, before proceed to indexing PCR. We also carried a negative control (buffer EBT) to control for contaminations.

For the indexing PCR of the first 50 samples we tested for 8, 9, 10 and 11 cycles of amplification. As we saw an increase in the concentration, we showed that at 11 cycles had not reached plateau. We chose 8 cycles to reduce the potential number of PCR duplicates. For the other 64 samples we performed 10 cycles in order obtain a better yield. In the cases where there was not enough PCR product for the multiplexing step we repeated the library amplification step. The results of the indexing PCR were verified on an agarose/TBE gel in order to observe the shift in the fragment distribution (126 bp more heavy). The final elution of the beads was cleaned up after indexing PCR was performed with water instead of buffer EBT. The rationale for this step was that, after multiplexing, the samples have to be concentrated on a SpeedVac machine (CentriVap DNA Concentrator, LABCONCO) and the use of EBT buffer will increase the salt concentration, which might affect the hybridization.

**Hybridization Experiment**

We performed three hybridization experiments using Agilent SureSelect custom 1M-feature capture microarrays. Experiment 1 included morphologically identified specimens from the same species that we used to design the probes (24 *T. brevignatha* + 26 juveniles) and served as a test run for the chip. Experiment 2 included specimens from the three closely related *green* species (15 *T. brevignatha* + 1 *T. macracantha* + 13 *T. waikamoi* + 23 juveniles). Finally, Experiment 3 had specimens from two *green* species (6 *T. macracantha* + 1 *T. waikamoi* + 5 juveniles).

In the multiplex step for each experiment we collected a total of 21 μg of DNA corresponding to equimolar amounts from all the libraries used. 20 μg went into the hybridization and 1 μg was stored as a pre-capture aliquot for positive and negative controls in qPCR assessment after the hybridization. The Post-Capture elution was concentrated using a SpeedVac (CentriVap DNA Concentrator, LABCONCO) until approximate 245 μl. We used an aliquot to test the number of cycles (16 and 18) for the whole library amplification. As there was an increase in concentration between 16 and 18 cycles, we decided to amplify the rest of the Post-Capture with 16 cycles as we could be sure that number of cycles was below PCR plateau. The Master mix consisted of: 31.1 μl of concentrated Post-Capture elution, 10 μl of Phusion buffer (5x), 1 μl of IS5 primer (10 μM), 1 μl IS6 primer (10 μM), 0.5 μl Phusion Hot Start High-Fidelity DNA Polymerase (2 U/μL) and dNTPs (25 mM each).

For Experiments 2 and 3 we initially tested for a smaller number of cycles in order to reduce the possibility of PCR duplicates. However we were not able to obtain any DNA in either 14 or 16 cycles. For Experiment 2 we ended up pooling the PCR products of 2 reactions of 20 cycles and 2 reactions of 21 cycles. While for Experiment 3 we pooled the PCR product of 2 reaction of 21 cycles and 2 reactions of 23 cycles.

The number of extra cycles that we performed for each sample was guided by the concentration measured by Qubit® 2.0 Fluorometer (Life Technologies) that we obtained for each case. The main problem with increasing the number of amplification cycles was the inevitable production of PCR duplicates, which decreases the sequencing depth of the genes of interest. The bioinformatics treatment that we used for the removal of PCR duplicates is explained in the section “Data preparation”.

**Post-Capture controls**

*Positive and Negative controls.* Following the protocol of [11], we used both positive and negative controls in the experiments to determine the global enrichment efficiency of the capture. While baits for the positive controls remained on the chip, we removed the baits for the negative control. For all controls we checked for the presence of duplicates or similar sequences. Using the partially annotated transcriptome of *T. brevignatha*, primers were designed to amplify 150 bp fragments. For this purpose we used the web-based program Primer 3 [12, 13]. Sequences for the primers are presented in Table S2. We chose the sequences Tb40119 and Tb9898 as a positive controls and a fragment of Histone 3 as a negative control. The primer sequences correspond to Tb40119.2, Tb9898.2 and H3.1.

To test the primers we used a PCR reaction mix of: 1 μl of each primer at 10 μM, 2 μl of AmpliTaq® (Life Technologies) buffer 10x, 0,5 μl of MgCl_2_ 25 mM, 11.7 μl H_2_O, 1 μl BSA 1x, 0.2 μl DreamTaq® (Thermo Scientific) and 1 μl of DNA extraction. The amplification profile started with 2 minutes at 95°C, followed by 40 replications of a cycle which started with 30 seconds at 95°C, then 30 seconds at 60°C and finally one minute at 72°C; there was an extra step of extension at 72°C for ten minutes. The PCR products were subsequently verified on an agarose/TBE gel.

*Enrichment and depletion*

For the qPCR validation, all the Pre and Post-Capture aliquots were diluted to a final concentration of 2 ng/μl. The master mix corresponded to: 2 μl of DNA (2 ng/μl), 6 μl of water, 10 μl of qPCR reaction mix, 1 μl Forward primer and 1 μl Reverse primer.

For each experiment we had two positive and one negative control. Those controls were tested in duplicates using as a template aliquots from: Pre-Capture, Post-Capture and qPCR Negative (no template). Reading the amplification plot we determined the shift in the number of cycles associated with the enrichment (Positive control) and depletion (Negative control).

A reduction in the number of cycles required to reach plateau in the Post-Capture for the positive control sequences is indicative of a success in the enrichment. In the same way, an increase in the number of cycles required to reach a plateau in the Post-Capture for the negative control is indicative of the success in the depletion of the non-specific sequences. It is not expected an absolute depletion of the non-captured sequences as it is known that the chip based approach captures a lot of unspecific fragments.

In Experiment 1 there was a shift of 5 cycles in both positive controls (Additional file 2; Figure S1a and S1n), which means that there was an enrichment of the captured sequences. Depletion was a little more than two cycles for the Negative control (Additional file 2: Figure S1c). The negative control for the qPCR starts enrichment at cycle 26, which is an expected result given the sensitivity of the technique. However, it does not affect the previous interpretation of our results, as the enrichment in the positive controls starts around the cycle 12. In all cases the replicates produced similar outcomes. In Experiment 2 the positive controls also showed a shift in approximately 5 cycles (Additional file 3: Figure S2a and S2b). The depletion in Experiment 2 is less pronounced than in Experiment 1. It is less than a full cycle (Additional file 4: Figure S3c). The negative control for the qPCR showed enrichment around cycle 26 (Additional file 3: Figure S2d). This is the same as in Experiment 1. In Experiment 2 the enrichment plots for the qPCR replicates have more variation than in Experiment 1, but it does not make any difference in the interpretation. In Experiment 3, the positive controls also showed a shift of approximately 5 cycles (Additional file 4: Figure S3a and S3b) and the negative control a shift of 1 cycle (Additional file 4: Figure S3c) Note that all the replicates, except Pre-Capture Tb40119, show overlapping curves. The negative control for the qPCR showed no enrichment (Additional file 3: Figure S2d).

*Bioanalyzer*

In order to ensure that the correct size distribution was sent to the sequencing facility, it was estimated with a Bioanalyzer (Agilent Technologies) for each experiment. We performed two replicates and one negative.

For Experiment 1 the fragment distribution ranged between 200 bp and 400 bp, with a peak in 267 bp (Additional file 5: Figure S4). There were no extra peaks at smaller or higher molecular weights. This implies that the size distribution was adequate for sequencing.

For Experiment 2 there is also a fragment distribution between 200 bp and 400 bp, with a peak of 245 bp (Additional file 6: Figure S5a). However, there was a second spike at 129 bp corresponding to primer dimers that were produced as a consequence of the high number of PCR cycles of the Post-Capture amplification. These fragments were eliminated with a bead cleaning. After this treatment the fragment distribution was adequate for sequencing (Additional file 6: Figure S5d).

The fragment distribution for Experiment 3 also ranged between 200 bp and 400 bp with a peak at 244 bp (Additional file 7: Figure S6a). But the amount of DNA recovered was very small in comparison to Experiment 1 and 2. Correlated with the difficulty in detecting DNA after whole library amplification. As a consequence of the large number of PCR cycles the same extra peak at 129 bp was detected. It was also eliminated by an extra bead-cleaning step, producing a fragment distribution amenable to sequencing (Additional file 7: Figure S6b). The bead cleaning for removing primer dimers and cleaning up product for sequencing was performed at the Vincent J. Coates Genomics Sequencing Laboratory at UC Berkeley.

**Analytical pipeline**

***Data preparation***

All the scripts used on steps 1 to 6 for data preparation are on the CGRL github web site (<https://github.com/CGRL-QB3-UCBerkeley/denovoTargetCapturePopGen>).

1.- *Merging left and right files*: Note that it is necessary to change the names into the same format as the genomic DNA reads. Script used: 1-pre-cleanup.pl.

2.- *Remove contaminants, duplicates and low complexity sequences*: The “libinfo file” is a tab-separated file with the equivalences of the bar codes and name of the specimens (it expects a header):

INDEX SPECIMEN

IndexXXX specimenXXX

indexYYY specimenYYY

The insert size (-e) was 185. The contaminant file (-c) consisted in a human and bacterial genomic data set to screen for contaminants.

The output files are: 1, 2, u, duplicates, contaminants and low complexity. Script used: 2-scrubReads_Nov12.pl.

3.- *kmeric assembly:* First, all the files 1, 2 and u for each library were combined with the respective 1, 2 and u files from the other libraries within the same species. It produced three combined files (1, 2 and u) for each species.

Then, we run ABySS [14] to create four different assemblies with a different kmer size each (k=23, 32, 45, 64). A sliding window approach was used to create the different kmers sizes and ABySS only saves the unique kmers into a library. After that, the program produces De Bruijn graphs with all the unique kmers. These graphs were used as a reference to define the contigs (these contigs have all the information of the polymorphism). All the individual reads of each library were mapped to these contigs, the script used was: [3-generateAssemblies_LOCAL.pl](https://github.com/MVZSEQ/transcriptome/blob/master/3-generateAssemblies_LOCAL.pl).

The result is a single FASTA file for each kmer size. These files are concatenated into a unique file.

5.- *Merge kmeric assemblies:* As a way to remove redundancy, the script [5-finalAssembly.pl](https://github.com/MVZSEQ/transcriptome/blob/master/5-finalAssembly.pl) takes information from all the kmer sizes to produce a new file with the best contigs.

6.- *Pseudo Reciprocal BLAST:* In order to find assemblies derived from targets we performed a Pseudo Reciprocal BLAST of the contigs produced in the Merged kmeric assembly against the sequences of the original target. This could be considered as an *in silico* repetition of the hybridization experiment. All the unspecific contigs will not be able to find a hit in the original target, which will eliminate them. It is important to mention that this step corresponds to a BLAST and not to an assembly, because the assembly algorithm would have problems with the intronic flaking regions next to each exon (100 bp approximate). The script used for this is: [6-pseudoReciprocalBlastToMakeFinalTargets.pl](https://github.com/MVZSEQ/Exon-capture/blob/master/6-pseudoReciprocalBlastToMakeFinalTargets.pl).

The output is the <intarget.fasta> file, which is equivalent to the original set of target sequences, but including the intronic flanking regions. This FASTA file will be used as a reference (-ref) for downstream analysis and as we do not have an ancestral reference to polarize the characters, it will be also use as an ancestral file (-anc). It means that the estimations of Site Frequency Spectrum (SFS) will correspond to folded SFS.

7.- *Mapping of each library:* For each library we mapped the reads present in the files 1, 2 and u. The average insert size of the libraries (-i) was 185 bp. For the other parameters we used the default settings. This step creates the bam files that will be used for the data analysis. The script used is: Alignment-by-novoalign.pl. It as a dependency the program NOVOALIGN ([www.Novocraft.com](http://www.Novocraft.com)).

8.- *Duplicate removal:* The elimination of duplicate sequences is always an important step in the data preparation. If this step is omitted the final coverage will be over estimated, which will negatively affect downstream analysis. This is especially relevant in the case of using genotype likelihood estimations. The program PICARD (<http://broadinstitute.github.io/picard/>) uses bam files as input to identify and eliminate duplicates.

9.- *Coverage estimations:* In order to have a realistic estimation, these calculations have to be done in the bam files after duplicate removal. To calculate coverage for each library we used the tool DEPTH in the program SAMTOOLS [15]. The following is the line of command to obtain the average per library:

>samtools depth -q 20 individual1.bam | awk '{sum+=$3} END { print "Average = ",sum/NR}'

***Basic evaluation of Exon capture performance***

The total amount of information yield from each experiment corresponded to: Experiment 1: 31 Giga bites, Experiment 2: 28 Giga bites and Experiment 3: 35 Giga bites. A total of 3,688 contigs were assembled from the capture experiments from an original target of 1,826 contigs. The higher number of assembled contigs is explained by the presence of non-targeted sequences and contaminants (removed on later stages of data analysis). The total number of bases captured was 1.4 Mega Bases from an original target of 1.7 Mega bases. The average coverage of the experiments before duplicate removal was 27.7x±4.9x, 27.8x±8.1x and 44.7x±9.0 for experiments 1, 2 and 3, respectively. The average coverage after duplicate removal with PICARD was 12.2x±1.6x, 3.8x±0.9x and 1.9x±0.3x for experiments 1, 2 and 3, respectively.

***Data analysis***

Given the low coverage of our data and its demonstrated high performance in middle coverage data sets, for the data analysis we used the program Analysis of Next Generation Sequencing Data (ANGSD). It can be downloaded from: <http://popgen.dk/wiki/index.php/ANGSD>. Before using ANGSD it is necessary to select the sites that will be used in the analysis. This selection requires applying several site-specific filters implemented in SNP Cleaner (<https://github.com/CGRL-QB3-UCBerkeley/denovoTargetCapturePopGen/blob/master/10-SNPcleaner>). After the allele frequency estimation based on genotype likelihood estimations has been done, it is possible to use the output files from ANGSD in other analysis. They are implemented in other programs as ngsTOOLS [16], SplitsTree V4.13.1 [17], NgsAdmix [18], PopGenTools (https://github.com/CGRL-QB3-UCBerkeley/denovoTargetCapturePopGen/blob/master/11-PopGenTools) and several R packages [19]. The analytical pipeline that we used is as follows:

1.- *VCF file, required for SNP Cleaner:* This file can be created with the program MPILEUP implemented in SAMTOOLS [15]. The input for this program corresponds to the reference file (FASTA file produced in step 7 of the “Data preparation”)

In case the version SNPcleaner231.pl is used, it would be necessary to create a Pileup file. That was not our case, as we used the version SNPcleaner02.pl.

2.- *SNP cleaner:* We used the version SNPcleaner02.pl. It requires the VCF file in the same folder as the program (https://github.com/CGRL-QB3-UCBerkeley/denovoTargetCapturePopGen/blob/master/10-SNPcleaner). We used a minimum coverage (-d) of 3x in 70% of the libraries (-k). Note the parameter –k is the actual number of libraries.

3.- *keep file:* One of the output of SNPCleaner is a bed file <*.bed>. It corresponds to a file with all the sites present on each contig that passed the filters. The file has three columns, which correspond to: contig, start position and end position. Because the program is looking sites, the file will look like this:

m.0001 0 1

m.0001 1 2

m.0001 2 3

…

The actual position of the site corresponds to the third column. In order to create the keep file we selected only the columns 1 and 3.

4.- *rf file:* The rt file corresponds to all the unique contigs that are present in the keep file. We generate it using the following command line:

>cat Name.keep | cut -f1 | sed "s/$/\:/g" | uniq > Name_rf

5.- *bam.filelist:* The bam.filelist is a text file with the paths to all the bam files to be used. To create it, we open the folder with all the bam files and used the following command line:

>find `pwd` *bam | grep "^/Home" | grep ".bam$" > bam_filelist.txt

6.- *ANGDS:* The details about how to run ANGSD can be found on the website of the program. To estimate the SFS and use it as an input for ngsTOOLS we used the following options: <-only_proper_pairs 0 -minMapQ 0 -minQ 20 -GL 1 -fold 1 -doSaf 1 -doMajorMinor 1 -doMaf 2 -doPost 1 -doGeno 32> We also tried several values for “-SNP_pval”, which corresponds to a filter that penalizes mostly the sites present in the first SFS category.

7.- *Site Frequency Spectrum (*.sfs):* We used the program emOptim2 and a *.saf file as input. Note that the *.saf file correspond to one of the outputs of ANGSD. For plotting the SFS in R, the first element of the file has to be deleted as it corresponds to the invariant sites.

8.- *Principal Component Analysis (PCA):* The first step is to create a Covariate file. For this we used the program ngsCOVAR present in the package ngsTOOLS [16]. The number of sites corresponds the number of lines in the *.mafs file (one of the outputs of ANGSD). Note that this file has a header, so the total number of sites is the number of lines minus one. The output can be plotted in R.

9.- *NgsAdmix*: In order to verify the most likely distribution of the samples given a fixed number of groups and detect admixture we used the program NgsAdmix [18]. The first step was to map all the specimens against the same reference (in this case we used *T. brevignatha*), which required generating new bam files and repeating the Mapping step with NOVOALIGN (Step 7 of “Data preparation”). Then, using those bam files (duplicates removed) we generate a Beagle file using ANGSD. The commands for generating the Beagle file and running NgsAdmix are on the website of the program (<http://www.popgen.dk/software/index.php/NgsAdmix>).

For each value of K, we performed 10 replications and plotted the one with the highest likelihood value that was repeated (Skotte *pers. comm.*). We tested the K values from 1 to 10.

To have an idea of the optimal value of K, we implemented the Evanno method [20] to select the optimal value of k, using an Excel spreadsheet. This implementation already exists for microsatellite data (STRUCTURE HERVASTER, [21]), but it does not exist for NGS applications. However, this was only used as a guide as NgsAdmix was not designed to find an optimal K value (Skotte *pers. comm.*).

The plotting of the results was done in R following a modified version of the code that appears on the website of the program. It was provided by one of the authors of NgsAdmix (Skotte *pers. comm.*).

10.- *SplitsTree*: In order to represent the phylogenetic relationships among the different populations of these three closely related species, we constructed an unrooted phylogenetic network using the program SplitsTree V4.13.1 [17]. This approach allows representing a collection of incompatible trees that are equally consistent with the data given a model of evolution [22]. This situation will be common in events of hybridization, horizontal gene transfer, recombination, and gene formation/duplication/loss. In particular, due to the known role of admixture in early speciation events [23, 24, 25], this method is particularly suitable for our system.

We took the clean 1, 2 and u files for each specimen and mapped them against the same reference (*T. brevignatha*). The new bam files were used to re-run ANGSD, but in this case we added a few more extra filters < -doGeno 2 -SNP_pval 0.1 –postCutoff 0.75 -geno_minDepth 3 -minInd 81 -doCounts 1 -doGlf 4 >. These filters were applied in order to obtain high quality data and remove sites with lower coverage (-geno_minDepth) than a determined cutoff (-minInd). In the case of our analysis we chose to keep sites with a minimum of 3x in at least 70% percent of the individuals.

The resulting geno file (*.geno) it is used as an input for [PopGenTools_2.80_New.pl](http://popgentools_2.80.pl/), this new version it is still not on the GitHub site. It was used to produce an *.adegenet file, which is an intermediate file for Splitstree V4.13.1. The line of command used was:

>perl ../PoulationGenomics/PopGenTools_2.80_New.pl Adegenet -g <genofile> -n <number of specimens> -s <number of sites. wc –l *.pos> -o saa.adegenet -h 1 –m 0.3

The option -m corresponds to the percentage of individuals with missing data for a particular site that is allows. Above that percentage the site was removed. A good recommendation is 0.3 (Bi *pers. comm.*). We used that filter to take care of missing data.

In the *.adegenet file columns correspond to the sites and the lines to the individuals. It was used as an input file for the R script splittrees_generator.docx (available upon request), which produces a file *.D. This file was used as an input for the script splittree.pl (available upon request) that generates a nexus file that can be opened by SplitsTree V4.13.1.

11.- *Genetic differentiation (Fst), Neutrality test (Tajima’s D) and Nucleotide diversity (π)*

*Fst calculations for two populations*: The first step was to run ANGSD for all the populations of interest. Then, we merged the files that contained all the used sites (*.pos) and selected only the unique sites. This can be accomplished with the following line of command:

>cat population1.saf.pos population2.saf.pos | sort | uniq -d > intersect.keep

After creating a file with all the common sites <intersect.keep>, we used the option FST on the program PopGenTools_2.76.pl in order to obtain do multiple comparisons across different populations. The file <intersect.keep>, is used on the –f option.

*Tajima’s D*: First, we estimated SFS and theta for each population. It cannot be the same SFS file used for Fst calculations, because for Tajima’s D has to be folded. As part of the options we had to include <–GL 1 –fold 1 –anc>. The –anc option corresponds to the “ancestral genome”. As we did not have an outgroup, we just used the same file as the reference (-ref). The result will be a file with the Tajima’s D for each contig. This distribution was plotted for each population. In order to get an exome-wide Tajima’s D we created a new theta file where all the sites were present in the same chromosome. In order to do that, we replaced all the values of the #Chromosome (column 1) for the same number. All of this was done with the script GlobalTajimaInput.pl (available upon request). Then we recompressed the file, and used the thetaStat program to estimate Tajima’s D [26].

*Nucleotide diversity (Pi)*: From Tajima’s D output file *.thetas.gz.pestPG we took the value of theta P (tP) and divided by the number of sites used on that population.

**Acknowledgements (extended version)**

The authors would like to acknowledge a large number of people and institutions that collaborated at different stages of this research. The fieldwork in Hawaiʻi was supported by Laura Arnold, Timothy Bailey, David Benítez, Katie Champlin, James Friday, Emory Griffin-Noyes, Faith Inman-Narahari, Darcey Iwashita, Raina Kaholoaa, Susan Kennedy, Jessie Knowlton, Rick Lapoint, Scott Laursen, Karl Magnacca, Elizabeth Morrill, Patrick O’Grady, Rita Pregana, Donald Price, David Rankin, William Roderick, Andrew Rominger, Karen Uy, Erin Wilson and the Kīpuka team.

The permit processing and access to different reserves and private land was possible thanks to Steve Bergfeld (DOFAW Big Island), Pat Bily (TNC) Maui, Tabetha Block (HETF), Shalan Crysdale (TNC Big Island), Lance DaSilva (DOFAW Maui), Danae Dean (Kahoma Ranch), Charmian Dang (NAR), Melissa Dean (HETF), Betsy Gagne (NAR), Elizabeth Gordon (HALE), Lisa Hadway (DOFAW Big Island), Paula Hartzell (Lanaʻi Resorts, LLC), Greg Hendrickson (Kealakekua Ranch), Mel Johansen (TNC Big Island), Pomaika‘i Kaniaupio-Crozier (Maui Land and Pinneapple), Cynthia King (DLNR), Peter Landon (NAR Maui), Rhonda Loh (HAVO), Russell Kallstrom (TNC Molokaʻi), Joey Mello (DOFAW Big Island), Ed Misaki (TNC Molokaʻi), Elliot Parsons (Puʻu Waʻawaʻa HETF), Lani Petrie (Kapapala Ranch), Shawn Saito (Parker Ranch), Joe Ward (Maui Land and Pinneapple) and Kawika Winter (Limahuli Botanical Garden).

We also appreciate the advices on lab and analytical work of Jacob Crawford, Peter Croucher, Emiliano Méndez, Rasmus Nielsen, Tyler Linderoth, Sonal Singhal, Line Skotte, Lydia Smith, LindLab, EvoLab. Barker DNA Sequencing Facility (UC Berkeley) and Vincent J. Coates Genomics Sequencing Laboratory at UC Berkeley (supported by NIH S10 Instrumentation Grants S10RR029668 and S10RR027303). Jonathan Price for discussions about the age of the wet forest on Leeward Big Island and Amy Vandergast for advice at the beginning of the project. George Roderick, Charles Marshall, Miquel Arnedo, the reviewers and, in particular, the editor, contributed with constructive comments to the manuscript.

DC’s PhD program was funded by a Fulbright/CONICYT fellowship and a researcher position on the Hawaiʻi NSF Dimensions of Biodiversity Project. The fieldwork on Hawaiʻi was funded by Graduate Research Allocation Committee (Integrative Biology dept. UC Berkeley), Summer Research Grant (Integrative Biology dept. UC Berkeley), Walker Grant (Essig Museum of Entomology), Sigma Xi grant and Research Grant Graduate Division UC Berkeley.

**References**

1.- Grabherr MG, Haas BJ, Yassour M, Levin JZ, Thompson DA, Amit I, Adiconis X, Fan L, Raychowdhury R, Zeng Q, Chen Z, Mauceli E, Hacohen N, Gnirke A, Rhind N, di Palma F, Birren BW, Nusbaum C, Lindblad-Toh K, Friedman N, Regev A. Full-length transcriptome assembly from RNA-seq data without a reference genome. Nat Biotechnol 2011;29:644-652.

2.- Croucher PJP, Brewer MS, Winchell CJ, Oxford GS, Gillespie RG. De novo characterization of the gene-rich transcriptomes of two color-polymorphic spiders, *Theridion grallator* and *T. californicum* (Araneae: Theridiidae), with special reference to pigment genes. *BMC Genomics* 2013:**14**:862.

3.- Huson DH, Auch AF, Qi J, Schuster SC. MEGAN analysis of metagenomic data. Genome Res 2007;17:377-86.

4.- Parra G, Bradnam K, Korf I. CEGMA: a pipeline to accurately annotate core genes in eukaryotic genomes. Bioinformatics 2007;**23**:1061-7.

5.- Tatusov RL, Fedorova ND, Jackson JD, Jacobs AR, Kiryutin B, Koonin EV, Krylov DM, Mazumder R, Mekhedov SL, Nikolskaya AN, Rao BS, Smirnov S, Sverdlov AV, Vasudevan S, Wolf YI, Yin JJ, Natale DA. The COG database: an updated version includes eukaryotes. *BMC* Bioinformatics 2004;4:41.

6.- Trifonov VA, Vorobieva NN, Rens W. Chapter 9 FISH With and Without COT1 DNA. In: Liehr T, editor. Fluorescence In Situ Hybridization (FISH) – Application Guide Heidelberg, Germany: Springer-Verlag; 2009. p. 99-109.

7.- Brewer MS, Cotoras DD, Croucher PJP, Gillespie RG. 2014 New sequencing technologies, the development of genomics tools, and their applications in evolutionary arachnology*.* J Arachnol 2014;42:1–15.

8.- Sanggaard KW *et al.* Spider genomes provide insight into composition and evolution of venom and silk. Nat Commun 2014;5:3765.

9.- Meyer M, Kircher M. Illumina sequencing library preparation for highly multiplexed target capture and sequencing. Cold Spring Harb Protoc; 2010

10.- Hodges E, Rooks M, Xuan Z, Bhattacharjee A, Benjamin Gordon D, Brizuela L, Richard McCombie W, Hannon GJ. Hybrid selection of discrete genomic intervals on custom-designed microarrays for massively parallel sequencing. Nat Protoc 2009;4:960-74.

11.- Bi K, Vanderpool D, Singhal S, Linderoth T, Moritz C, Good JM. Transcriptome-based exon capture enables highly cost-effective comparative genomic data collection at moderate evolutionary scales. BMC Genomics 2012;13:403.

12.- Koressaar T, Remm M. Enhancements and modifications of primer design program Primer3. Bioinformatics 2007;23:1289-91.

13.- Untergasser A, Cutcutache I, Koressaar T, Ye J, Faircloth BC, Remm M, Rozen SG. 2012 Primer3 - new capabilities and interfaces. Nucleic Acids Res 2012;40:e115.

14.- Simpson JT, Wong K, Jackman SD, Schein JE, Jones SJ, Birol I. ABySS: a parallel assembler for short read sequence data. *Genome Res.* 2009;**19**:1117-23.

15.- Li H, Handsaker B, Wysoker A, Fennell T, Ruan J, Homer N, Marth G, Abecasis G, Durbin R, 1000 Genome Project Data Processing Subgroup. The Sequence alignment/map (SAM) format and SAMtools. Bioinformatics 2009;25:2078-9.

16.- Fumagalli M, Vieira FG, Linderoth T, Nielsen R. ngsTools: methods for population genetics analyses from next-generation sequencing data. Bioinformatics 2014;30:1486-7.

17.- Huson DH, Bryant D. Application of phylogenetic networks in evolutionary studies. Mol Biol Evol 2006;23:254-67.

18.- Skotte L, Korneliussen TS, Albrechtsen A. Estimating individual admixture proportions from next generation sequencing data. Genetics 2013;195:693-702.

19.- R Development Core Team. R: a language and environment for statistical computing. 2008 R Foundation for Statistical Computing. Vienna, Austria. Accessed:: http://www.R-project.org.

20.- Evanno G, Regnaut S, Goudet J. 2005 Detecting the number of clusters of individuals using the software STRUCTURE: a simulation study. Mol Ecol 2005;14:2611–20.

21.- Earl DA. 2011 Structure harvester v0.6.1. Accessed: http:// taylor0.biology.ucla.edu/struct_harvest/

22.- Bandelt HJ, Dress AWM. 1992 A canonical decomposition theory for metrics on a finite set. Adv Math 1992;92:47–105.

23.- Seehausen O. Hybridization and adaptive radiation. Trends Ecol Evol 2004;19:198–207.

24.- Martin SH, Dasmahapatra KK, Nadeau NJ, Salazar C, Walters JR, Simpson F, Blaxter M, Manica A, Mallet J, Jiggins CD. 2013 Genome-wide evidence for speciation with gene flow in *Heliconius* butterflies. Genome Res 2013;23:1817–28.

25.- Rius M, Darling JA. How important is intraspecific genetic admixture to the success of colonising populations? Trends Ecol Evol 2014;29:233-242.

26.- Korneliussen TS, Moltke I, Albrechtsen A, Nielsen R. Calculation of Tajima’s D and other neutrality test statistics from low depth next-generation sequencing data. BMC Bioinformatics 2014;14:289.

**Table S1: Collecting sites**

| **Island** | **Volcano** | **Site** | **GPS coordinate** |
| --- | --- | --- | --- |
| Maui | East Maui (Haleakalā) | Upper Waikamoi TNC | N 20°46’41.60’’  W 156°13’43.21’’ |
|  |  | Lower Waikamoi TNC | N 20°48’24.22’’  W 156°15’18.02’’ |
|  |  | Koʻolau FR (Near Cramp) | N 20°45'39.70''  W 156°08'34.57'' |
|  |  | Koʻolau FR (Day 2) | N 20°45'26.85''  W 156°08'35.56'' |
|  |  | Kīpahulu Valley (Up) | N 20°43'11.06''  W 156°05'15.74'' |
|  |  | Kīpahulu Valley (Other) | N 20°42'48.83''  W 156°06'12.36'' |
|  | West Maui | Puʻu Kukui (Upper) | N 20°54’52.04”  W 156°35’31.84” |
|  |  | Puʻu Kukui (Lower) | N 20°54’56.73”  W 156°35’35.51” |
| Lanaʻi | Lanaʻi | Munro trail | N 20°48'28.37''  W 156°52'03.96'' |
| Big Island | Hualālai | Honoaula FR (Makahi St. entry) | N 19°43’07.96’’  W 155°56’57.34’’ |
|  | Mauna Kea | Laupāhoehoe HETF (H) | N 19°55’50.43”  W 155°17’20.48” |
|  |  | Laupāhoehoe HETF  (High) | N 19°54’57.62”  W 155°18’22.91” |
|  |  | Laupāhoehoe HETF (Maulua trail. Plot 32, Transect 31) | N 19°53’54.53”  W 155°18’46.35” |
|  | Mauna Loa | Kīpuka 15 | N 19°40’18.15”  W 155°20’18.36” |
|  |  | Kīpuka 18 | N 19°40’27.03”  W 155°19’59.45” |
|  |  | Kīpuka 5+37+23 | N 19°39’50.19”  W 155°21’09.72” |
|  |  | Forest (f2) | N 19°39’57.39”  W 155°21’09.91” |
|  |  | Kaʻu FR (Kapapala access) | N 19°20’41.82”  W 155°28’03.14” |
|  |  | Kona Hema TNC | N 19°12’49.41”  W 155°49’44.48” |
|  |  | Puʻu Makaʻala NAR (Army Road) | N 19°33'03.36''  W 155°13'51.54'' |

TNC: The Nature Conservancy; NAR: Natural Area Reserve; FR: Forest; HETF: Hawaiʻi Experimental Tropical Forest; HAVO: Hawaiʻi Volcanoes National Park

**Table S2: Primer sequences for Positive and Negative controls**

| **Gene** | **Primer name** | **Sequence** |
| --- | --- | --- |
| H3 | H3.1_L | GAAGCAGTTGGCAACCAAGG |
|  | H3.1_R | GAAGCTCGGTGGACTTCTGG |
| Tb9898 | Tb9898.2_L | CGGAAAAGAATGTGCTGTGC |
|  | Tb9898.2_R | CAGCACACATGAAGCGATCC |
| Tb40119 | Tb40119.2_L | ACGGGACTCTCCTGATGTCG |
|  | Tb40119.2_R | TGAGTGTGAGGGCACCTGAG |

**Table S3: Nucleotide diversity (**π) **and Neutrality test (Tajima’s D)**

| **Population** | π | **Tajima’s D** |
| --- | --- | --- |
| *T. brevignatha*, Maui | 0.0025 | -1.10 |
| *T. brevignatha*, Big Island | 0.0022 | -2.29 |
| *T. macracantha*, Lanaʻi | 0.0028 | -1.16 |
| *T. macracantha*, Maui | 0.0041 | -1.81 |
| *T. waikamoi,* East Maui | 0.0028 | -1.71 |
| *T. waikamoi*, West Maui | 0.0026 | -1.49 |

**Table S4: Genetic differentiation (Fst) between species**

|  | *T. brevignatha,* Big Island | *T. brevignatha,* Maui | *T. macracantha*  (Lanaʻi + Maui) | *T. waikamoi*  (East Maui + West Maui) |
| --- | --- | --- | --- | --- |
| *T. brevignatha,* Big Island | - | 0.74 | 0.74 | 0.69 |
| *T. brevignatha,* Maui | - | - | 0.69 | 0.64 |
| *T. macracantha*  (Lanaʻi + Maui) | - | - | - | 0.35 |

Global Fst = 0.66

**Figure S1: qPCR controls Experiment 1.** a Positive control (Tb9898), b Positive control (Tb40119), c Negative control (H3), d qPCR negative control. Orange: Post-capture, Blue: Pre-capture.

**Figure S2: qPCR controls Experiment 2.** a Positive control (Tb9898), b Positive control (Tb40119), c Negative control (H3), d qPCR negative control Experiment 2 and 3. Light blue: Post-capture, Blue: Pre-capture, Red: qPCR negative control Experiment 2, Orange: qPCR negative control Experiment 3.

**Figure S3: qPCR controls Experiment 3.** a Positive control (Tb9898), b Positive control (Tb40119), c Negative control (H3). Yellow: Post-capture, Orange: Pre-capture.

**Figure S4: Bioanalyzer read of the fragment distribution of the whole library after amplification (Experiment 1).**

**Figure S5: Bioanalyzer read of the fragment distribution of the whole library after amplification (Experiment 2).** a Before beads clean up, note the extra spike at 129 bp. b After beads clean up, the spike at 129 bp was removed.

**Figure S6: Bioanalyzer read of the fragment of the whole library after amplification (Experiment 3).** a Before beads clean up, note the extra spike at 129 bp. b After beads clean up, the spike at 129 bp was removed.

**Figure S7: Average Likelihood (10 independent replicates) vs K.** The graph does not include K=1. See Evanno *et al*. 2005

**Figure S8: Principal Component Analysis of all the specimens.** a PC1 vs PC3 and b PC2 vs PC3. The shapes represent the species assignation based in the locality given previous literature. Circles: *T. brevignatha*; Squares: *T, macracantha*; Triangles: *T. waikamoi* and Rhombus corresponds to a new locality that has not been surveyed until this study. The colours correspond to the identification based in morphology. Calypso: *T. brevignatha*; Dark green: *T, macracantha*; Light green: *T. waikamoi* and Grey: juveniles or specimens that could not be identified with certainty. Note that the two groups of *T. brevignatha* correspond with the Maui and Big Island populations.

**Figure S9: Principal Component Analysis of *T. macracantha* and its sympatric population of *T. waikamoi* (Kipahulu Valley)**

**Figure S10: Principal Component Analysis of *T. brevignatha* and its sympatric population of *T. waikamoi* (Lower Waikamoi)**

**Figure S11: NgsAdmix runs.** a K=2, b K=3, c K=4, d K=6, e K=8, f K=9 and g K=10. The specimens are separated by localities. The localities correspond to: PKK (Puʻu Kukui), UWKM (Upper Waikamoi), KIPA (Kīpahulu Valley), KOO (Koʻolau), LANI (Lanaʻi), LWKM (Lower Waikamoi), LPP (Laupāhoehoe), KIPK (Kīpuka), PMK (Puʻu Makaʻala), KAU (Kaʻu), KH (Kona Hema) and HNLA (Honoaula).

**Figure S12: Gene by gene Tajima’s D.** a Comparison of *T. brevignatha*, Big Island; *T. brevignatha*, Maui; *T. macracantha* and *T. waikamoi*. b Comparison of the two populations of *T. macracantha* (Lanaʻi and Maui) and c Comparison of the two populations of *T. waikamoi* (East Maui and West Maui).
