# Supplementary figures and images for: Co-occurrence of ecologically similar species of Hawaiian spiders reveals critical early phase of adaptive radiation
Source: BMC Evol Biol. 2018 Jun 19;18:100. doi: 10.1186/s12862-018-1209-y (PMC6009049; doi:10.1186/s12862-018-1209-y)

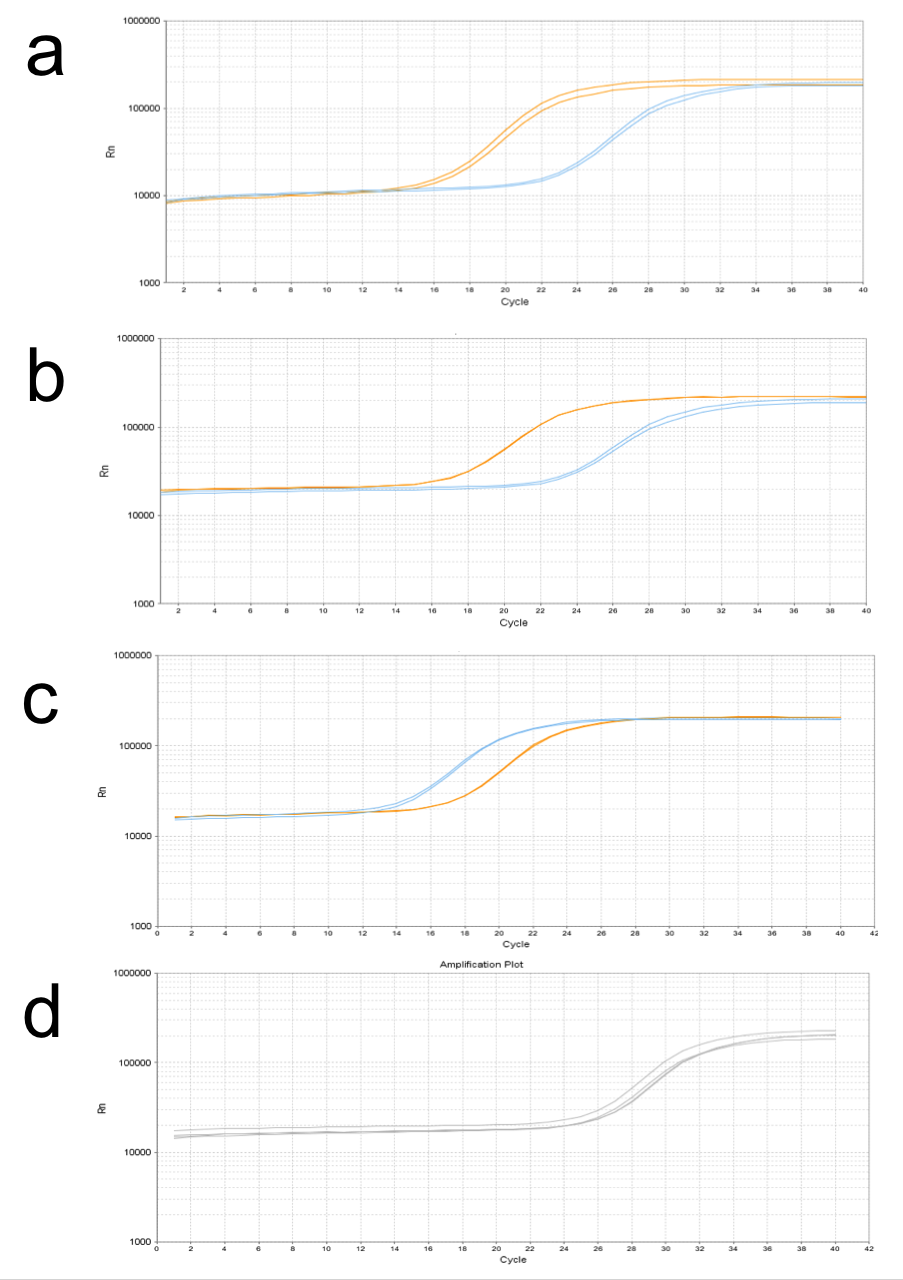

Supplement: Supplementary file 2 — Figure S1. qPCR controls Experiment 1. (TIFF 4517 kb) [file 12862_2018_1209_MOESM2_ESM.tiff]

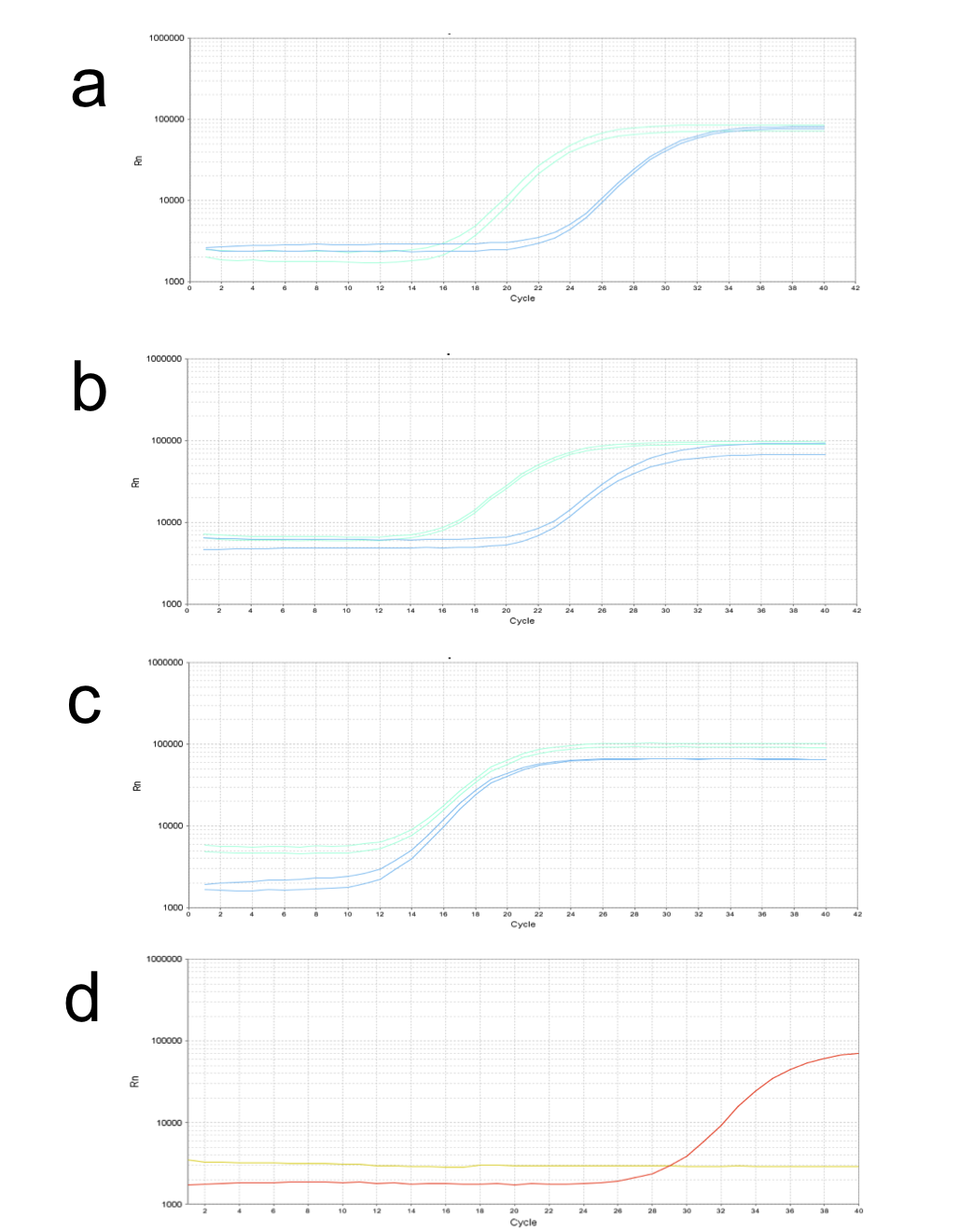

Supplement: Supplementary file 3 — Figure S2. qPCR controls Experiment 2. (TIFF 5307 kb) [file 12862_2018_1209_MOESM3_ESM.tiff]

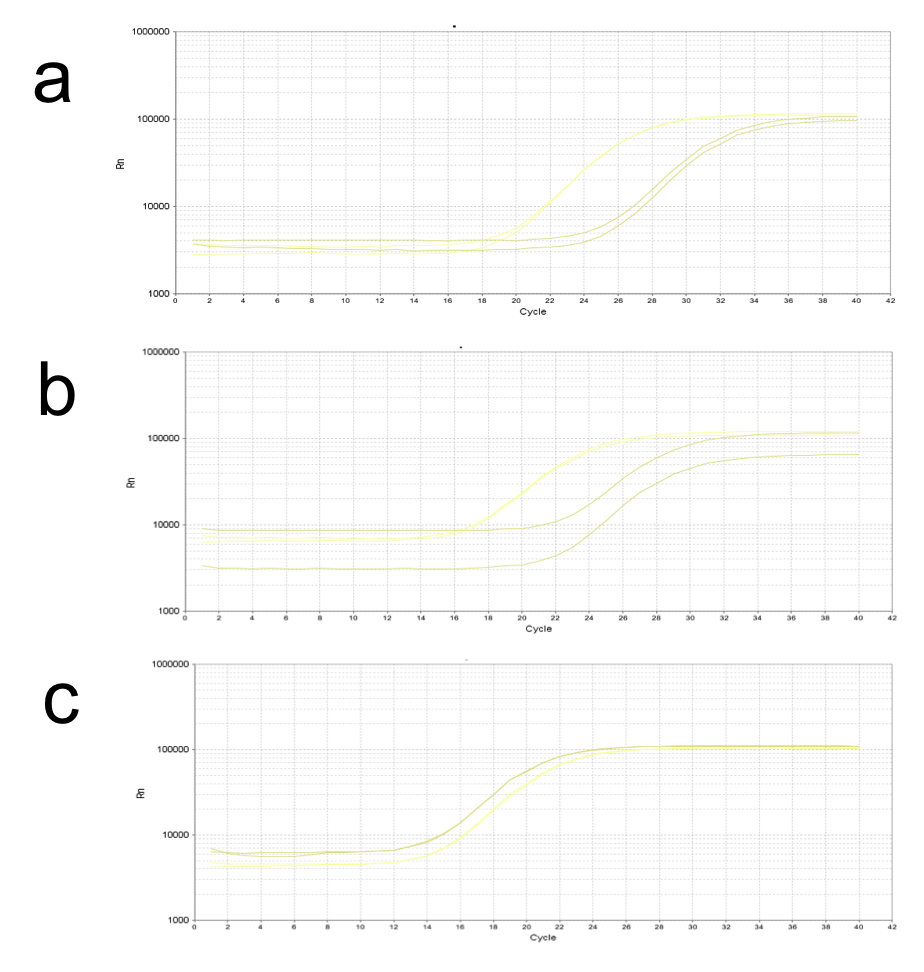

Supplement: Supplementary file 4 — Figure S3. qPCR controls Experiment 3. (TIFF 3510 kb) [file 12862_2018_1209_MOESM4_ESM.tiff]

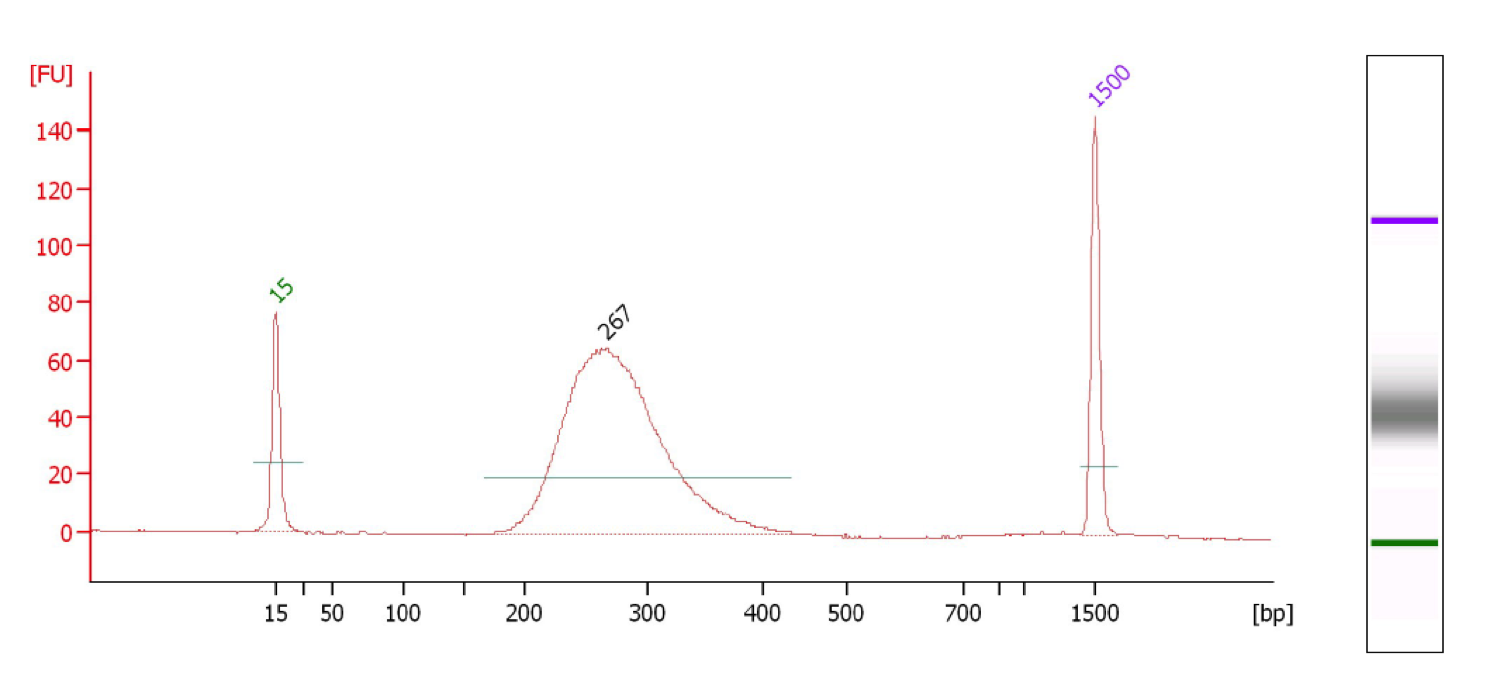

Supplement: Supplementary file 5 — Figure S4.Bioanalyzer read of the fragment distribution of the whole library after amplification (Experiment 1). (TIFF 3975 kb) [file 12862_2018_1209_MOESM5_ESM.tiff]

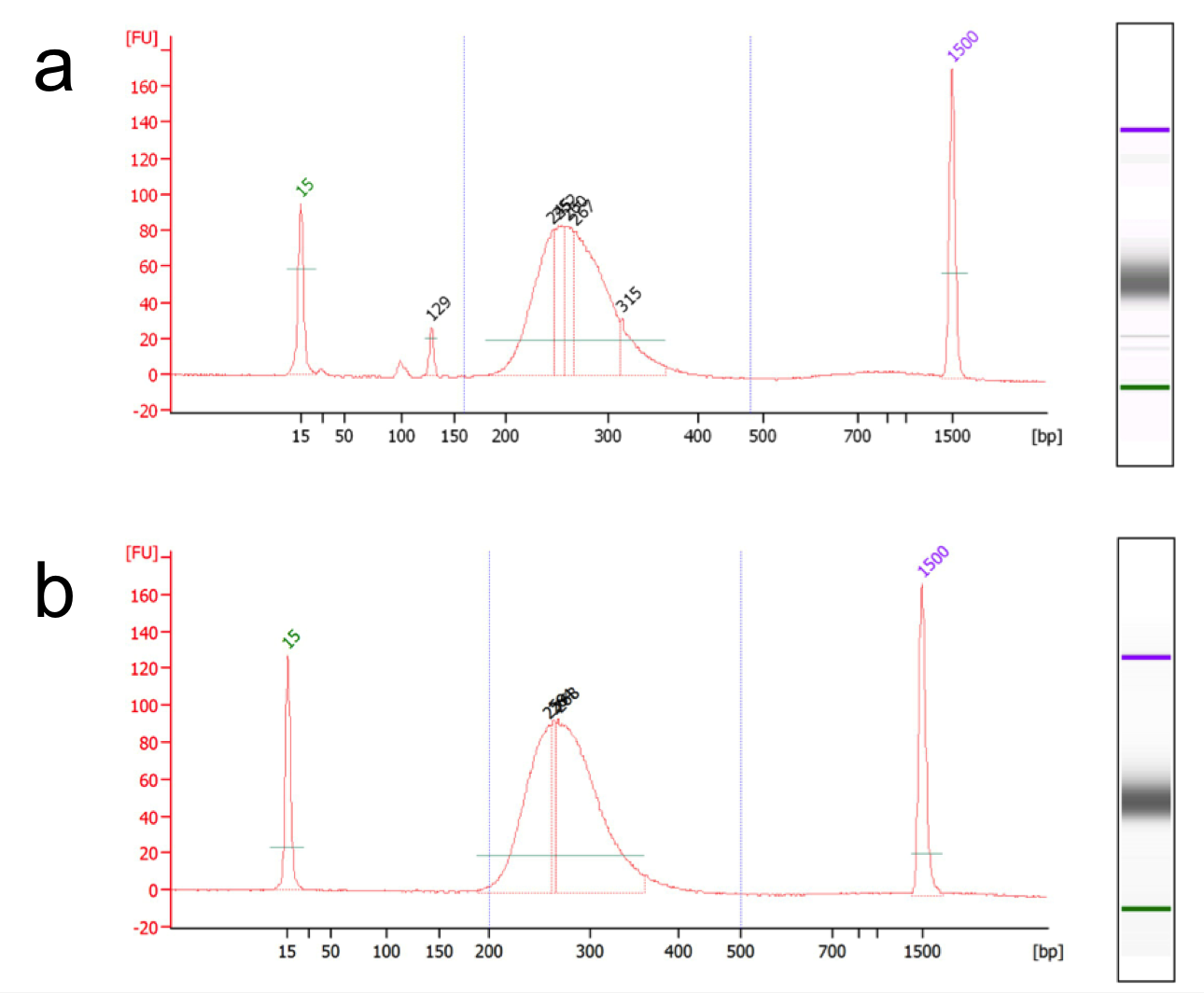

Supplement: Supplementary file 6 — Figure S5. Bioanalyzer read of the fragment distribution of the whole library after amplification (Experiment 2). (TIFF 5364 kb) [file 12862_2018_1209_MOESM6_ESM.tiff]

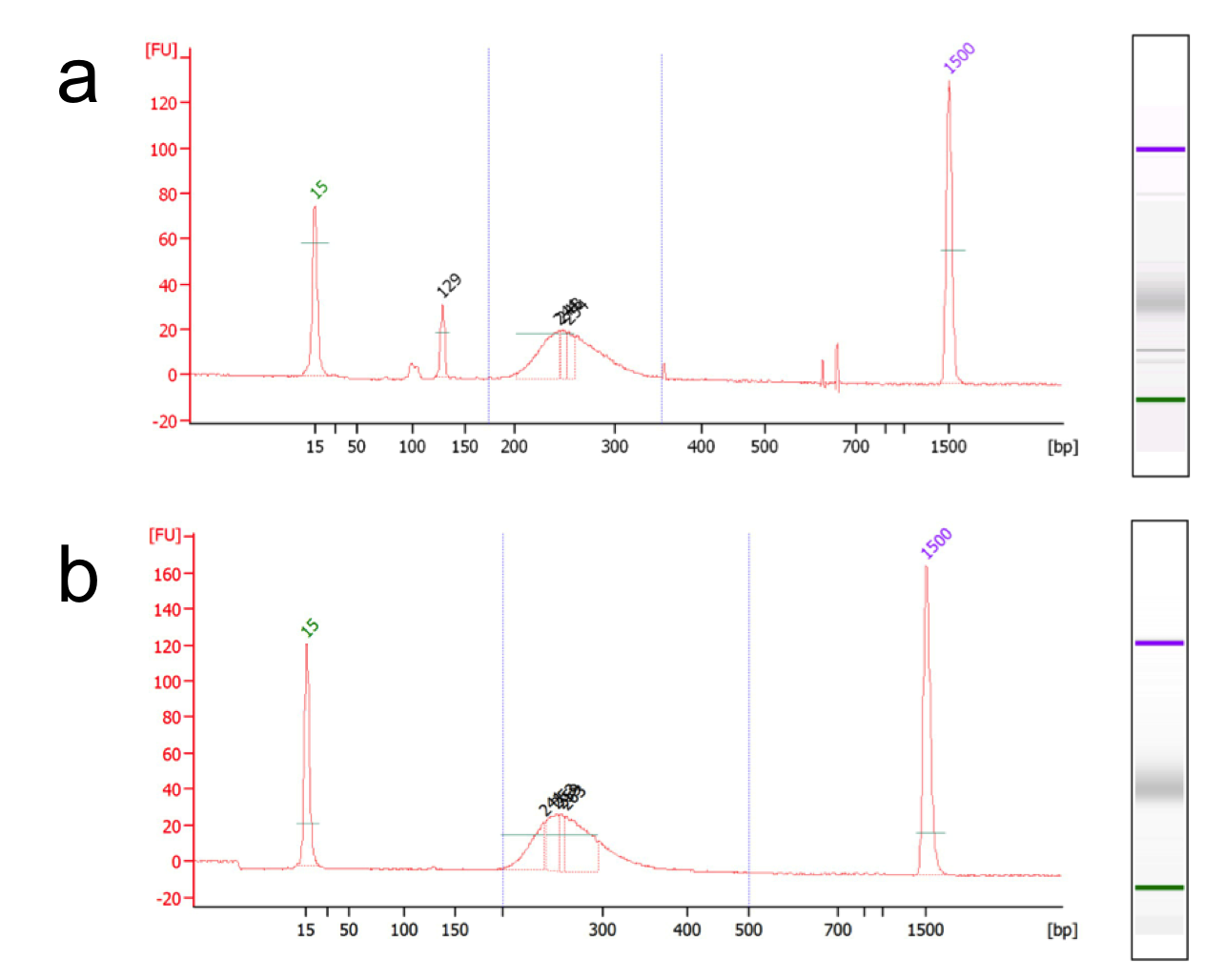

Supplement: Supplementary file 7 — Figure S6. Bioanalyzer read of the fragment of the whole library after amplification (Experiment 3). (TIFF 5162 kb) [file 12862_2018_1209_MOESM7_ESM.tiff]

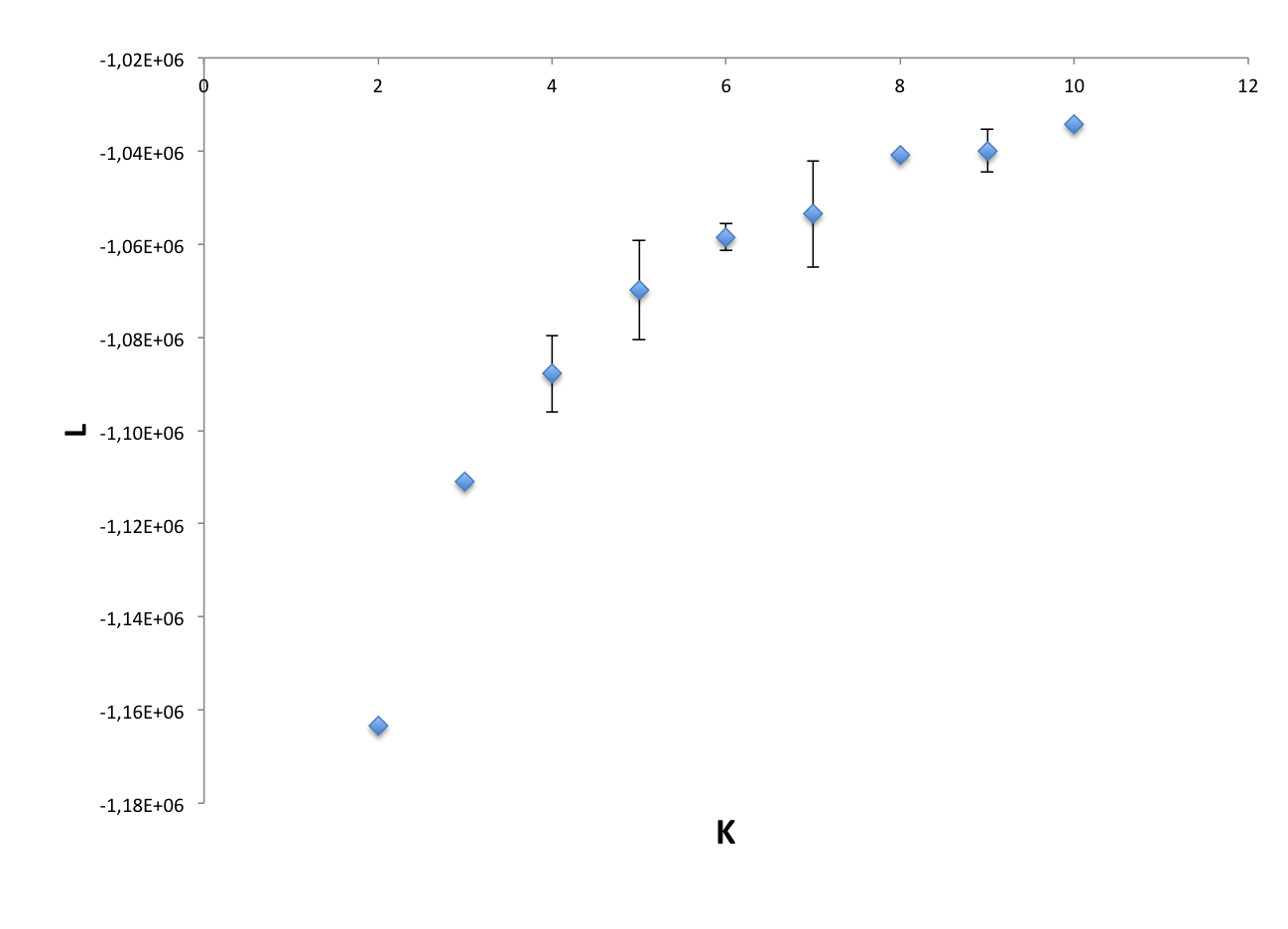

Supplement: Supplementary file 8 — Figure S7. Average Likelihood (10 independent replicates) vs K. (TIFF 4748 kb) [file 12862_2018_1209_MOESM8_ESM.tiff]

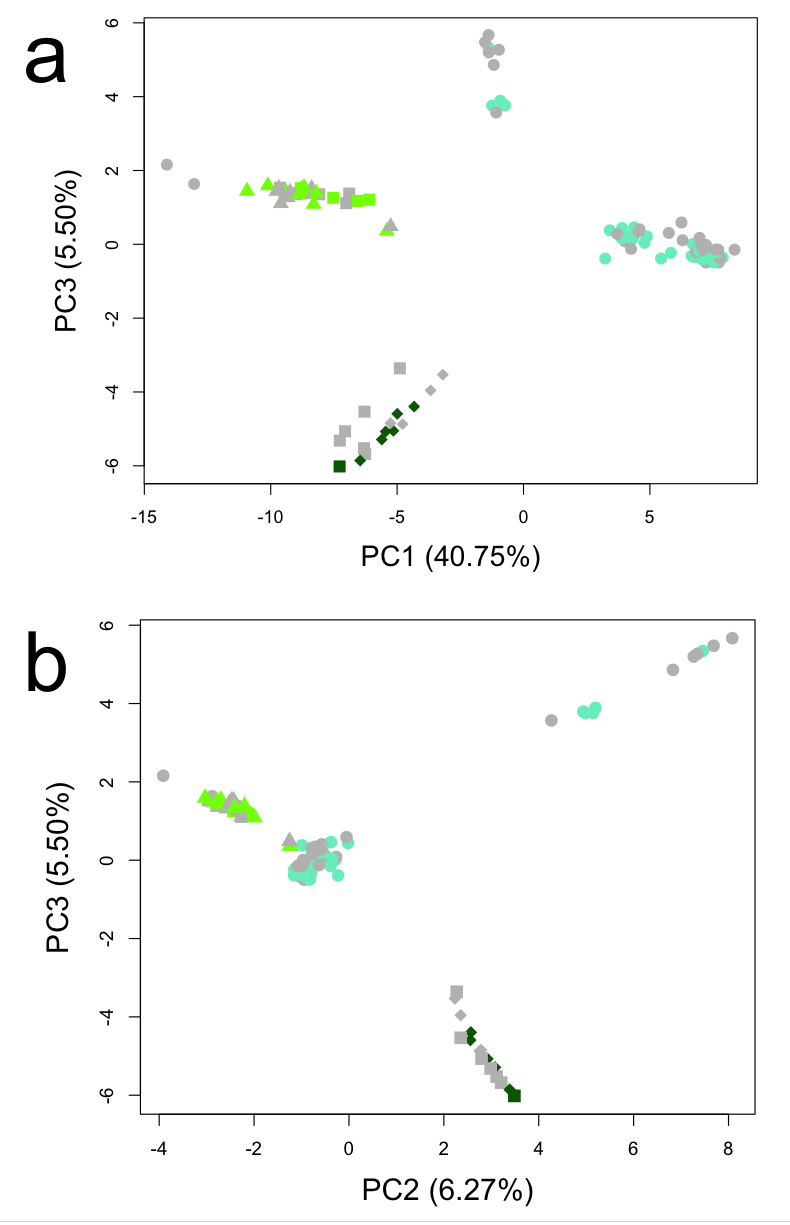

Supplement: Supplementary file 9 — Figure S8. Principal Component Analysis of all the specimens. a PC1 vs PC3 and b PC2 vs PC3. (TIFF 3782 kb) [file 12862_2018_1209_MOESM9_ESM.tiff]

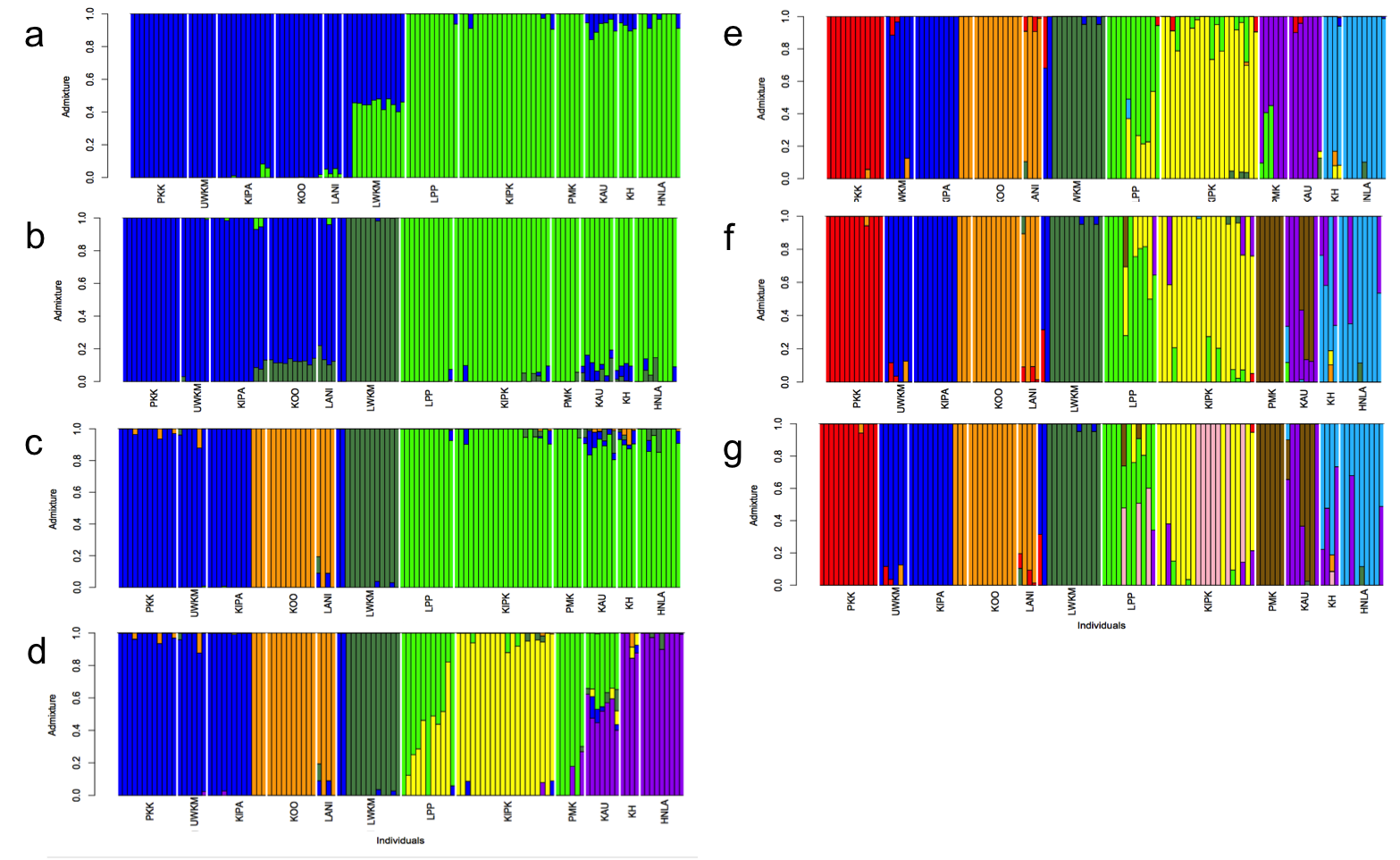

Supplement: Supplementary file 10 — Figure S11. NgsAdmix runs. a K=2, b K=3, c K=4, d K=6, e K=8, f K=9 and g K=10. (TIFF 5897 kb) [file 12862_2018_1209_MOESM10_ESM.tiff]

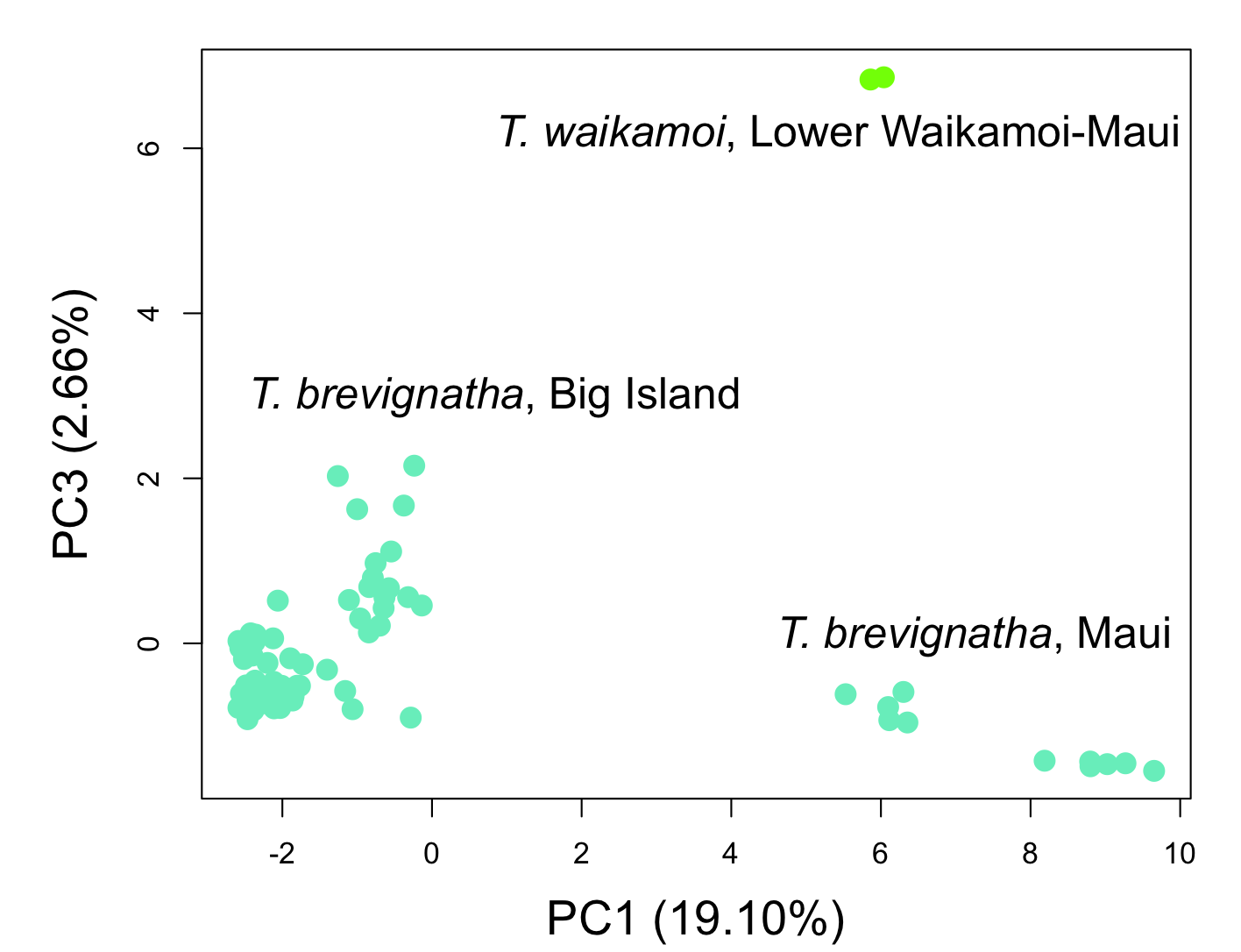

Supplement: Supplementary file 11 — Figure S10. Principal Component Analysis of T. brevignatha and its sympatric population of T. waikamoi (Lower Waikamoi). (PNG 114 kb) [file 12862_2018_1209_MOESM11_ESM.png]

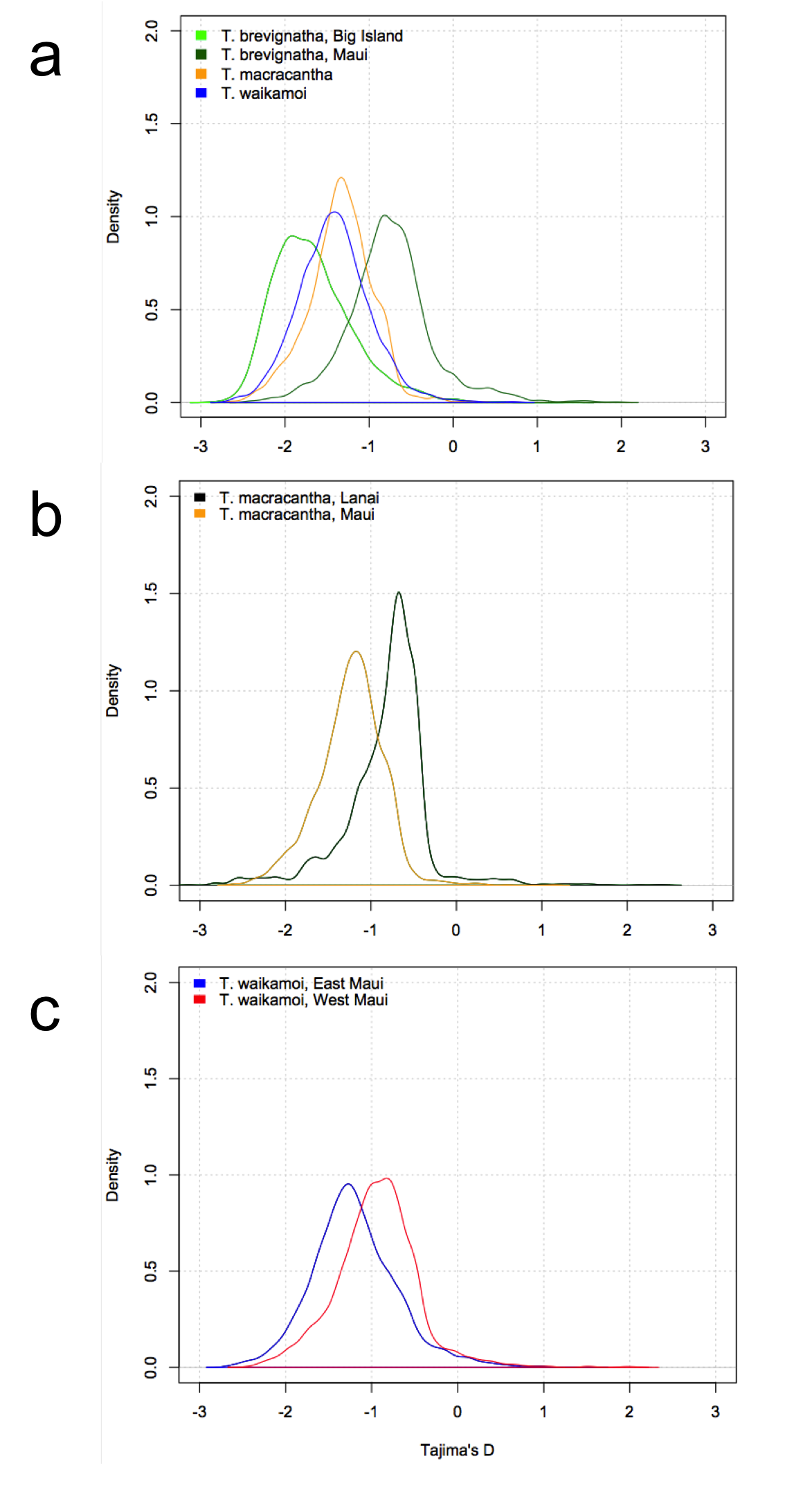

Supplement: Supplementary file 12 — Figure S12. Gene by gene Tajima’s D. (TIFF 9982 kb) [file 12862_2018_1209_MOESM12_ESM.tiff]

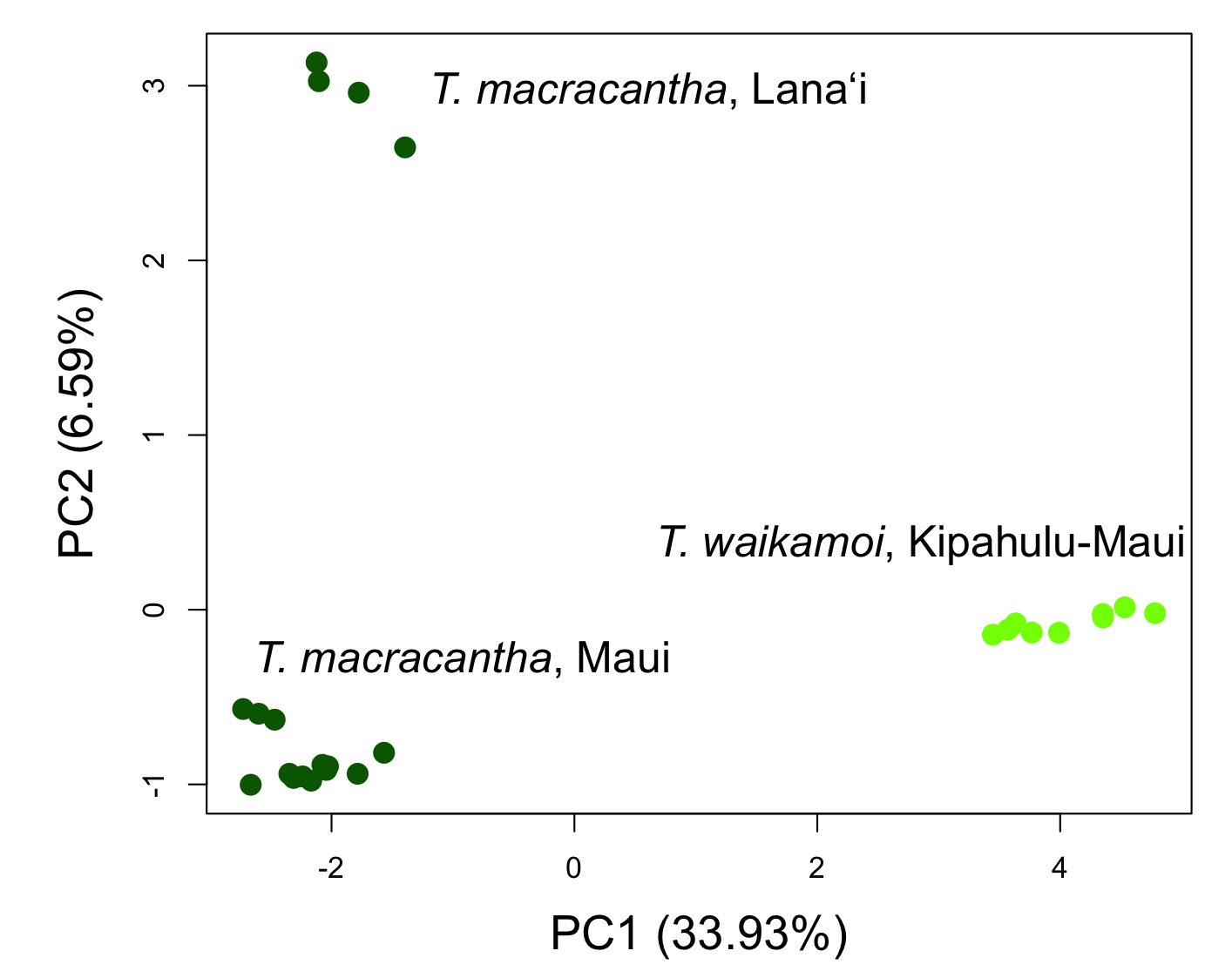

Supplement: Supplementary file 13 — Figure S9. Principal Component Analysis of T. macracantha and its sympatric population of T. waikamoi (Kipahulu Valley). (TIFF 6195 kb) [file 12862_2018_1209_MOESM13_ESM.tiff]
